# Hybrid Pd_0.1_Cu_0.9_Co_2_O_4_ nano-flakes: a novel, efficient and reusable catalyst for the one-pot heck and Suzuki couplings with simultaneous transesterification reactions under microwave irradiation

**DOI:** 10.3389/fchem.2024.1496234

**Published:** 2024-10-30

**Authors:** Ashok Raj Patel, Gurupada Maity, Tanmay K. Pati, Laksmikanta Adak, Christopher L. Cioffi, Subhash Banerjee

**Affiliations:** ^1^ Department of Chemistry, Guru Ghasidas Vishwavidyalaya, Bilaspur, India; ^2^ Department of Physics, School of Basic and Applied Science, Galgotias University, Greater Noida, India; ^3^ Department of Chemistry, Rensselaer Polytechnic Institute, Troy, NY, United States; ^4^ Department of Chemistry, Indian Institute of Engineering Science and Technology, Howrah, India

**Keywords:** spinel-type catalyst, nano-flake material, Mizoroki-Heck reaction, Suzuki coupling reaction, transesterification, microwave irradiation, recyclable catalyst, cross-coupling reactions

## Abstract

We report the fabrication of a novel spinel-type Pd₀.₁Cu₀.₉Co₂O₄ nano-flake material designed for Mizoroki-Heck and Suzuki coupling-cum-transesterification reactions. The Pd₀.₁Cu₀.₉Co₂O₄ material was synthesized using a simple co-precipitation method, and its crystalline phase and morphology were characterized through powder XRD, UV-Vis, FESEM, and EDX studies. This material demonstrated excellent catalytic activity in Mizoroki-Heck and Suzuki cross-coupling reactions, performed in the presence of a mild base (K₂CO₃), ethanol as the solvent, and microwave irradiation under ligand-free conditions. Notably, the Heck coupling of acrylic esters proceeded concurrently with transesterification using various alcohols as solvents. The catalyst exhibited remarkable stability under reaction conditions and could be recycled and reused up to ten times while maintaining its catalytic integrity.

## Introduction

Transition-metal-catalyzed cross-coupling reactions, namely, Mizoroki-Heck and Suzuki reactions, have gained recognition for both their utility and versatility in the construction of carbon-carbon bonds (Miyaura and Buchwald, 2002; Diederich and Stang, 2008; Negishi, 2011; Johansson Seechurn et al., 2012; De Meijere et al., 2013), which has widespread applications in the synthesis of biologically and pharmaceutically important scaffolds (Heck, 1979; Miyaura and Suzuki, 1995; Beletskaya and Cheprakov, 2000; Yin and Liebscher, 2007; Buchwald, 2008). Palladium was the first transition metal to be used as a catalyst for key organic reactions on an industrial level (Matos and Soderquist, 1998). Historically, homogeneous palladium catalysts in the form of metal salts with phosphines, *N*-heterocyclic carbenes (NHCs), and other organic ligands have been widely used in catalyzing cross-coupling reactions (Suzuki, 1999; Maureen, 2004; Jiang et al., 2007; Wu et al., 2010b; Wu et al., 2010a). However, growing economic and environmental concerns of homogeneous palladium catalysts have kickstarted research into the heterogenization of these catalysts. Researchers aim to create catalysts that maintain high catalytic activity while addressing economic and environmental concerns by immobilizing palladium on various inorganic and organic support materials. However, catalysts containing transition metals other than palladium, such as copper (Babu et al., 2013; Liwosz and Chemler, 2013; Gurung et al., 2014; Basnet et al., 2016; Tang et al., 2018; Budiman et al., 2019), cobalt (Asghar et al., 2017; Ludwig et al., 2020), or nickel (Gøgsig et al., 2012; Ramgren et al., 2013), have also been used to conduct various cross-coupling reactions. In recent years, spinels have gained recognition as active catalysts for organic transformations (Jagadeesh et al., 2013; Payra et al., 2016b; Payra et al., 2016a; Payra et al., 2018; Anke et al., 2019; Dong et al., 2019; Patel et al., 2020b; Ghazzy et al., 2022; Patel et al., 2022). These materials, also known as perovskites, are binary and ternary mixed metal oxides composed of mixed-valence transition metals, with a general formula of AB₂O₄, where A and B represent different metal cations. The presence of two mixed-valence metal cations facilitates electron transport between multiple transition metal cations, requiring relatively low activation energy (Jadhav et al., 2016; Kuang et al., 2016). Recently, spinel oxide-supported palladium catalysts such as PdAl_2_O_4_(Kannan, 2017), Pd/Fe_3_O_4_(Baran and Nasrollahzadeh, 2019), Pd/NiFe_2_O(Borhade and Waghmode, 2011), Pd/ZnFe_2_O_4_ (Singh et al., 2013), PdCuFe_2_O_4_ (Tong et al., 2016), PdCoFe_2_O_4_ (Senapati et al., 2012) have been reported to catalyze various cross-coupling reactions. Similarly to other spinels, Co_3_O_4_ adopts a normal spinel structure, consisting of Co^2+^ at tetrahedral sites and Co^3+^ at octahedral sites (Gao et al., 2016). In addition to the high activity, spinels provide additional benefits including low cost, ease of preparation, and high stability (Hamdani et al., 2010), Furthermore, the electrocatalytic efficiency of Co_3_O_4_ can be enhanced by the incorporation of additional metal ions (M = Zn, Cu, Ni, Mg, Fe, and Pd) into the oxide (Wu and Scott, 2011; Grewe et al., 2013; Rosen et al., 2013; Grewe et al., 2014; Liu et al., 2014; Kumar and Srivastava, 2020). The cobalt cation is partially substituted by a transition metal cation, which occupies the octahedral sites, while Co. occupies both the tetrahedral and octahedral sites, which in turn forms an inverse spinel structure (Liu et al., 2016). The use of cobalt catalysts, particularly CuCo_2_O_4_ have been reported in oxidation of alcohols (Jiang et al., 2019) and in the oxidative aza-coupling of amines. (Patel A. R. et al., 2020).

Transesterification is a classic organic reaction that involves the conversion of one ester into another through the exchange of alkoxy groups between an alcohol and the ester. Esters represent one of the most important functional groups found in polymers, agrochemicals, natural products, and biological systems, thereby making them widely applicable as key intermediates and/or protecting groups in organic transformations. (Larock, 1989; Otera and Nishikido, 2009; Nguyen et al., 2012). Transesterification reactions are widely used in organic synthesis and chemical industries (Otera, 1993; Grasa et al., 2004; Otera, 2004) as well as in polymer industries (Capelot et al., 2012) and biodiesel synthesis (Hindryawati et al., 2014; Lam et al., 2019). Recently, transesterification reactions that utilize diverse catalysts such as Lewis acids (Bosco and Saikia, 2004; Sheng and Kady, 2009), organic and inorganic bases (Jagtap et al., 2008; Watson et al., 2008; Sridharan et al., 2010), and *N*-heterocyclic carbenes (Grasa et al., 2002; Nyce et al., 2002; Singh et al., 2004; Zeng et al., 2009) have also been reported. However, there is currently no known methodology for performing one-pot cross-coupling reactions combined with transesterification.

**SCHEME 1:**
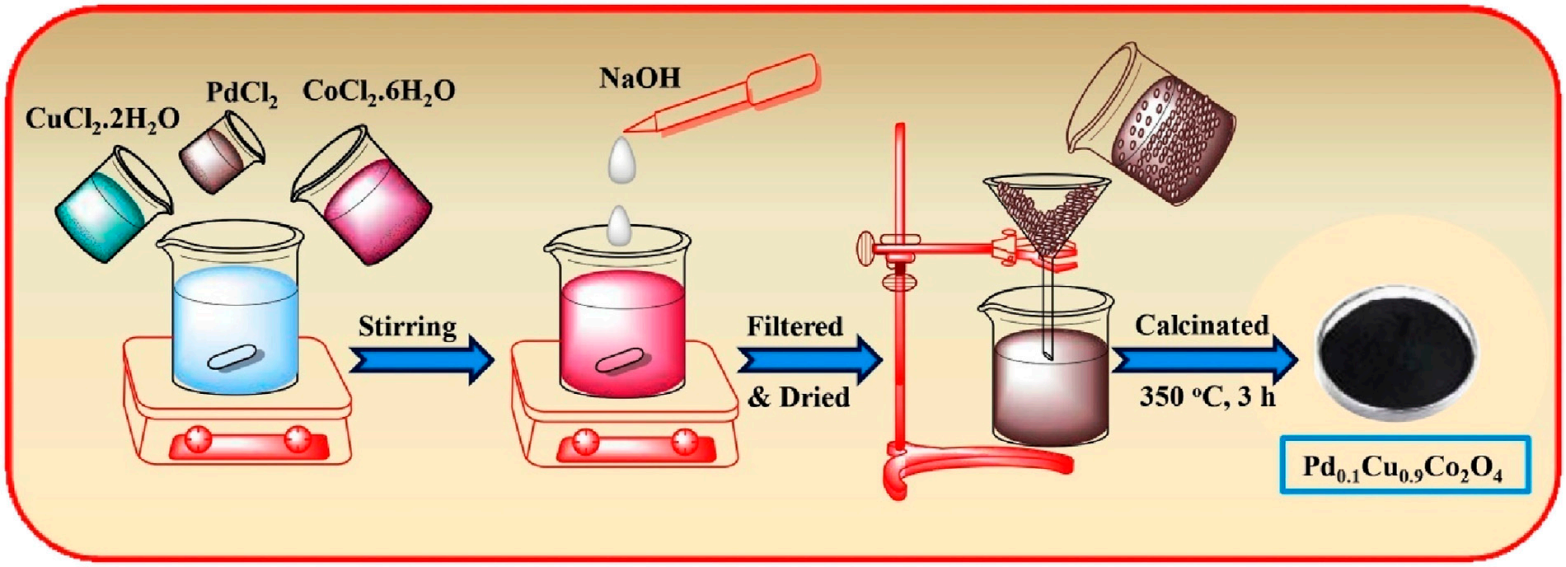
Schematic representation of Pd_0.1_Cu_0.9_Co_2_O_4_ preparation via co-precipitation method.

**SCHEME 2:**
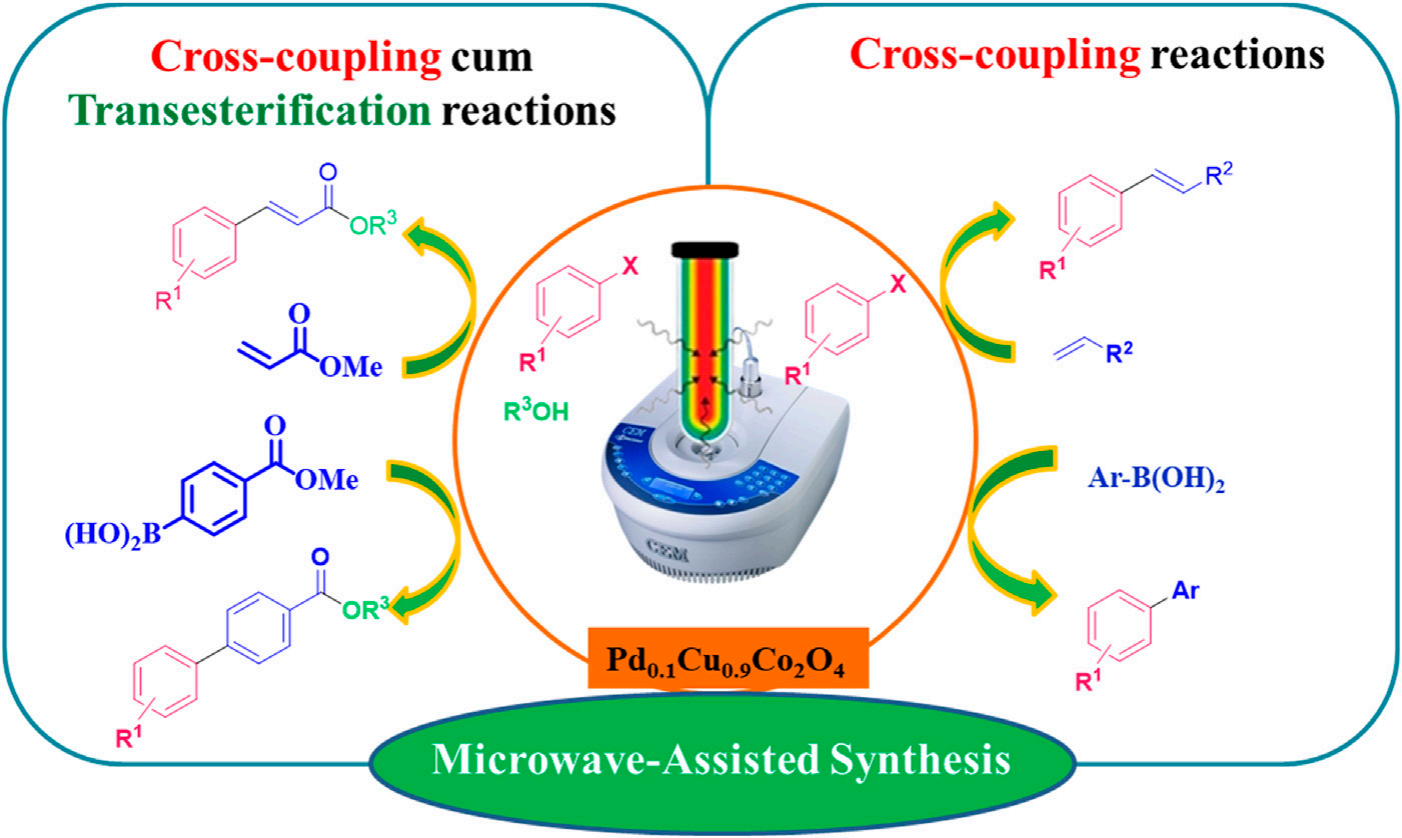
Microwave-assisted Heck and Suzuki coupling with concomitant transesterification.

As part of our ongoing efforts to develop novel transition metal-catalyzed reactions (Pati et al., 2018; Pati et al., 2020; Pati et al., 2024) and green synthetic methodologies using heterogeneous nanomaterials (Banerjee and Saha, 2013; Banerjee, 2015; Saha et al., 2015; Saha et al., 2017a; Saha et al., 2017b; Saha et al., 2018; Patel et al., 2019a; Patel et al., 2019b; Saha et al., 2019) we report the synthesis of hybrid Pd₀.₁Cu₀.₉Co₂O₄ spinel nano-flakes (Scheme 1), which effectively catalyze Mizoroki-Heck and Suzuki coupling reactions along with concomitant transesterification in a one-pot process. This occurs under ligand-free microwave irradiation conditions using an alcohol solvent (see Scheme 2).

## Result and discussion

Firstly, we synthesized CuCo₂O₄ and Pd-doped CuCo₂O₄ using a simple co-precipitation method. The CuCo₂O₄ was prepared following a previously reported procedure (Sudha et al., 2019), while the Pd-doped CuCo₂O₄ was synthesized by doping an appropriate amount of palladium into the CuCo₂O₄ structure (details in ESI).

**FIGURE 1:**
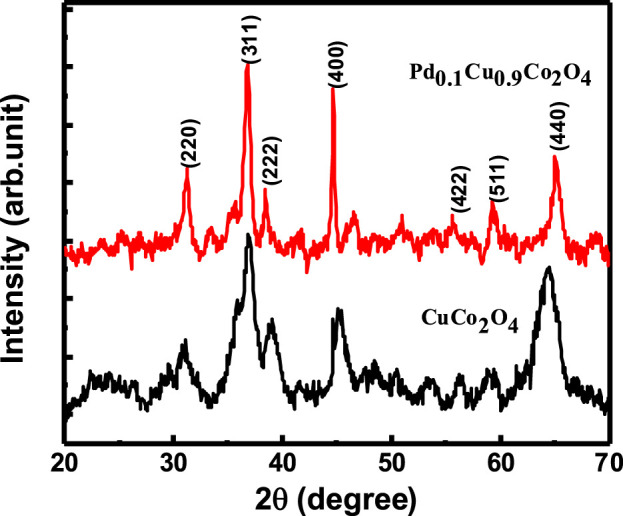
XRD of CuCo_2_O_4_(black) and Pd_0.1_Cu_0.9_Co_2_O_4_(Red).

To investigate the crystalline form of the samples, X-ray powder diffraction (XRD) measurements were performed. The XRD patterns of the CuCo₂O₄ and Pd₀.₁Cu₀.₉Co₂O₄ samples are presented in Figure 1. For the CuCo₂O₄ sample, diffraction peaks were observed at 2θ values of 18.77°, 31.08°, 36.86°, 38.66°, 44.63°, 56.45°, 59.30°, and 65.55°, corresponding to the (111) (220) (311) (222) (400) (422) (511), and (440) planes, respectively.

The diffraction peaks observed correspond to the polycrystalline cubic spinel phase of CuCo₂O₄ (JCPDS Card No. 01–1155). The Pd₀.₁Cu₀.₉Co₂O₄ sample exhibits diffraction peaks at the same 2θ values as the CuCo₂O₄ sample, confirming that Pd is fully doped into the Cu site without forming any impurity phases (Bikkarolla and Papakonstantinou, 2015; Patel A. R. et al., 2020). The crystallite sizes (D) for both the pure and Pd-doped samples were calculated using the Debye–Scherrer formula ( $D=\frac{0.9\lambda }{\beta \cos\theta }$ , where λ = 1.54 Å and β is FWHM) and they are found to be 14 nm and 10 nm for CuCo_2_O_4_ and Pd_0.1_Cu_0.9_Co_2_O_4_, respectively.

**FIGURE 2:**
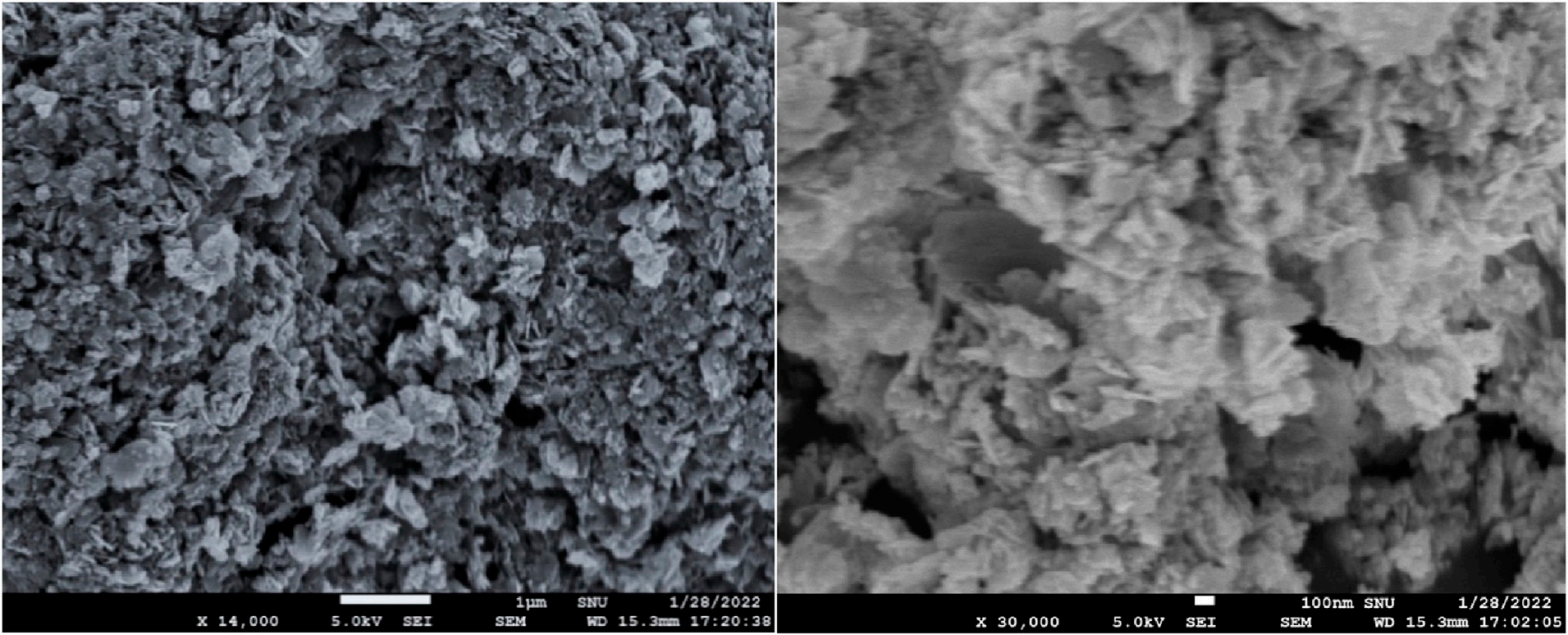
FESEM image of **(A)** CuCo_2_O_4_ and **(B)** Pd_0.1_Cu_0.9_Co_2_O_4_.

The morphology of the Pd₀.₁Cu₀.₉Co₂O₄ sample was examined using field emission scanning electron microscopy (FESEM). Figure 2 presents the FESEM image, revealing the formation of a flake-like structure in the material. The average nano-flake size ranges from 760 nm for CuCo₂O₄ to 205 nm for Pd₀.₁Cu₀.₉Co₂O₄, indicating that the Pd-doped samples have a higher surface area compared to the pure CuCo₂O₄ samples.

**FIGURE 3:**
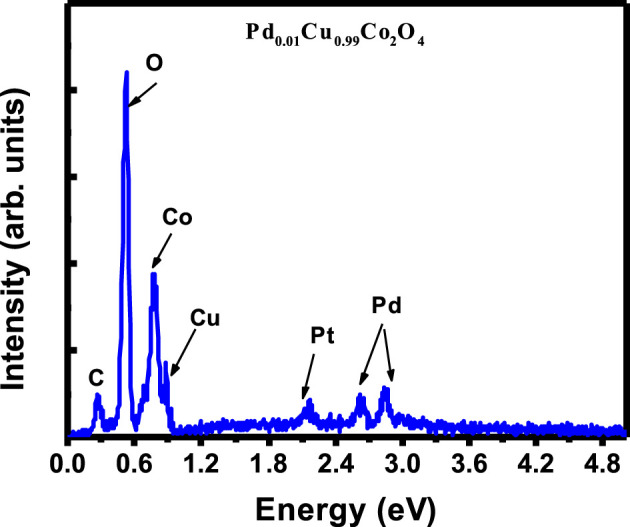
EDX spectra of Pd0.1Cu0.9Co2O4 material.

The elemental composition and purity of the Pd₀.₁Cu₀.₉Co₂O₄ sample were determined using energy-dispersive X-ray spectroscopy (EDX). The EDX spectrum, shown in Figure 3, confirms the presence of dispersive peaks corresponding to the elements C, O, Co., Cu, Pd, and Pt (the latter due to the Pt coating applied during SEM measurements). The absence of dispersive peaks for other elements, within the statistical limits of detection, indicates the high purity of the Pd₀.₁Cu₀.₉Co₂O₄ material. (Patel A. R. et al., 2020).

**FIGURE 4:**
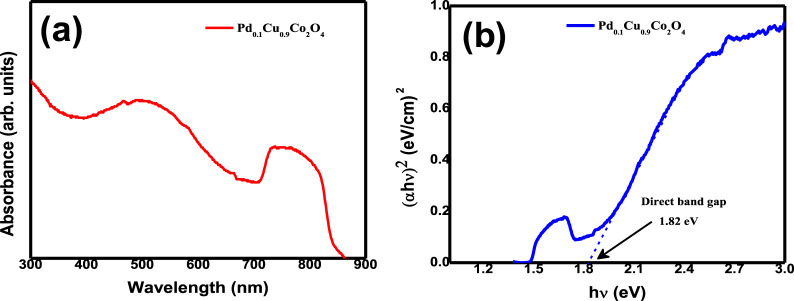
**(A)** UV-Vis spectrum of Pd₀.₁Cu₀.₉Co₂O₄ nano-flakes and **(B)** Tauc’s plot for the estimation of direct optical band gap for Pd₀.₁Cu₀.₉Co₂O₄ nano-flakes.

Additionally, the optical properties of the Pd₀.₁Cu₀.₉Co₂O₄ sample were investigated using UV–Vis spectroscopy. Figure 4 shows the UV–Vis absorbance spectra of the as-prepared Pd₀.₁Cu₀.₉Co₂O₄ nano-flakes, which exhibit a broad absorption range spanning both the UV and visible regions. Two distinct absorption bands were observed at 500 nm and 750 nm. The band gap was determined using Tauc’s relation: αhν = C (hν−E.g.,) n\alpha h\nu = C(h\nu - E_g)^∧^nαhν = C(hν−E.g.,) n, where hνh\nuhν represents the photon energy, EgE_gEg is the optical band gap, and CCC is the band tailing parameter. For direct allowed transitions, nnn was set to 2. Figure 4B presents the Tauc’s plot used to estimate the direct optical band gap of the Pd₀.₁Cu₀.₉Co₂O₄ sample, which was found to be 1.82 eV.

The catalytic activity of well-characterized Pd₀.₁Cu₀.₉Co₂O₄ nano-flakes was next evaluated in cross-coupling reactions. We began with the Heck coupling reaction of one-iodo-4-nitrobenzene (1) and acrylonitrile as a model system. When a mixture of one-iodo-4-nitrobenzene (1.0 mmol), acrylonitrile (1.5 mmol), K₂CO₃ (2.0 mmol), and Pd₀.₁Cu₀.₉Co₂O₄ nano-flakes (4 mol %) was stirred in DMF at 100°C, a 30% yield of (E)-3-(4-nitrophenyl) acrylonitrile (2) was obtained after 10 h (entry 1, Table 1).

**TABLE 1:** Optimization of reaction condition for Heck cross-coupling reaction^a^.

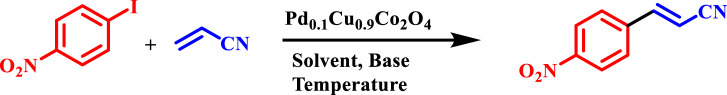						
Entry	Solvent	Base	Catalyst (mole %)	Temp. (^o^C)	Time	Yield (%)
1	DMF	K_2_CO_3_	4	100	10 h	30
2	DMF-H_2_O(2:1)	K_2_CO_3_	4	100	10 h	45
3	H_2_O	K_2_CO_3_	4	100	12 h	20
4	EtOH-H_2_O	K_2_CO_3_	4	Reflux	6 h	45
5	EtOH	K_2_CO_3_	4	Reflux	6 h	60
6	EtOH	K_2_CO_3_	8	Reflux	6 h	75
7	EtOH	K_2_CO_3_	12	Reflux	6 h	76
8	EtOH	NaOH	8	Reflux	6 h	27
9	EtOH	Na_2_CO_3_	8	Reflux	6 h	60
10	EtOH	KOH	8	Reflux	6 h	75
11	EtOH	K_2_CO_3_	8	MW^b^	10 min	>99
12	EtOH	K_2_CO_3_	4	MW^b^	10 min	98
13	EtOH	K_2_CO_3_	2	MW^b^	10 min	87
14	EtOH	K_2_CO_3_	4	MW^b^	5 min	76

^a^ Conditions:1-iodo-4- nitrobenzene (1.0 mmol), acrylonitrile (1.2 mmol), Base (2.0 equivalents), solvent (2.0 mL), and catalyst. Unless otherwise stated.

^b^ MW, Microwave irradiation conditions at 50 W, 100°C.

In microwave-assisted reactions, the solvent absorbs microwave energy through dielectric heating, rapidly increasing the temperature and speeding up the reaction. Polar solvents, which have a higher dipole moment, absorb microwaves more efficiently compared to nonpolar solvents with zero or low dipole moments. This enhances reaction rates and selectivity, enabling superheating that improves yields and reaction outcomes under conditions not achievable with conventional heating. We then proceeded to optimize the reaction conditions, starting with the screening of various solvents (entries 1–5, Table 1). Ethanol (EtOH) emerged as the optimal solvent, yielding a 60% product yield in 6 h (entry 5, Table 1). Further optimization involved varying the base, catalyst amount, and reaction time. Increasing the catalyst amount to 8 mol % improved the yield (entry 6, Table 1), but further increases in catalyst quantity did not enhance the yield (entry 7, Table 1). Among the bases tested, K₂CO₃ proved to be the most effective (entries 8–10, Table 1).

Finally, we conducted the model reaction under microwave (MW) irradiation, which offers several advantages over conventional heating methods. These include significantly reduced reaction times, selective and direct heating of reactants and reagents without heating the reaction vessel, improved yields, and reduced by-product formation (Payra et al., 2016b; Roberts and Strauss, 2005; Kappe and Dallinger, 2006; Patel et al., 2021).

Initially, when the reaction was conducted under microwave irradiation at 50 W and 100°C for 10 min using 8 mol % of catalyst, a significant improvement in yield (100% conversion, 99% yield) was achieved using 2.0 equivalents of K₂CO₃ in 1 mL of ethanol (entry 11, Table 1). A similar conversion was observed when the catalyst amount was reduced to 4 mole% (entry 12, Table 1). However, further reducing the catalyst to 2 mol% led to a slight decrease in yield (entry 13, Table 1). Additionally, shortening the reaction time to 5 min resulted in a reduced yield of 76% (entry 14, Table 1). Therefore, for 1 mmol of one-iodo-4-nitrobenzene, the optimized conditions for the model Heck coupling reaction were determined to be 4 mol% of Pd₀.₁Cu₀.₉Co₂O₄ nano-flakes in ethanol under microwave irradiation (50 W, 100°C, 10 min) (entry 12, Table 1).

Next, the scope of this methodology was explored under optimized reaction conditions by reacting various conjugated alkenes with aryl halides, following a general experimental procedure (see ESI for details). Notably, when methyl acrylate was used instead of acrylonitrile, the cross-coupling reaction led to transesterification when ethanol (EtOH) was used as the solvent. Encouraged by this finding, we investigated the cross-coupling of aryl halides with methyl acrylate in the presence of different alcohols as solvents. The results, summarized in Table 2, show that the Heck coupling reaction with methyl acrylate resulted in 100% transesterification when ethanol, n-propanol, or n-butanol was used (entries 1–4, 6, 9, Table 2). However, when propyl acrylate or butyl acrylate was used as the alkene, only the straight cross-coupling product was observed, with no transesterification occurring (entries 10–11, Table 2).

**TABLE 2:** Pd_0.1_Cu_0.9_Co_2_O_4_ NFs-catalyzed Heck coupling and concomitant transesterification reactions.

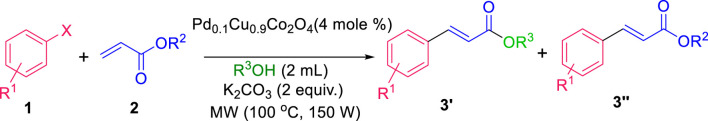						
Entry	R^1^	X	R^2^	Solvent (R^3^OH)	Chemoselectivity 3' : 3''	Yield (%)
1	H	I	Me	EtOH	100 : 0	96
2	OMe	I	Me	EtOH	100 : 0	97
3	H	I	Me	^n^PrOH	100 : 0	92
4	H	Br	Me	^n^PrOH	100 : 0	88
5	NO_2_	I	Me	^n^PrOH	90 : 10	92
6	OMe	I	Me	^n^PrOH	100 : 0	89
7	H	I	Me	^n^BuOH	70 : 30	97
8	H	Br	Me	^n^BuOH	70 : 30	90
9	NO_2_	I	Me	^n^BuOH	100 : 0	90
10	NO_2_	I	^n^Pr	EtOH	0 : 100	84
11	NO_2_	I	^n^Bu	EtOH	0 : 100	86

Conditions: aryl iodide (1.0 mmol), acrylate (1.2 mmol), K_2_CO_3_ (2.0 equiv.), alcohol solvent (2.0 mL), and catalyst 4 mole %, MW, 150 Watt, 80^o^C, 5 min.

Both aryl iodides and bromides reacted efficiently with various alkenes, such as acrylonitrile, methyl acrylate, and butyl acrylate, under the optimized conditions. Aryl iodides reacted faster than their bromide counterparts, likely due to the weaker C–I bond compared to the C–Br bond, which results in better leaving group ability for iodides, leading to higher yields with iodo-analogues. Additionally, we investigated the electronic effects of aryl halides on yield and reaction time with this catalytic system. Groups such as–NO₂ and–OMe on the aryl halides were well tolerated and enhanced the reaction rate. The reaction scope is detailed in Table 3. When acrylic acid was used, the Pd₀.₁Cu₀.₉Co₂O₄ nano-flakes catalyst facilitated the cross-coupling reaction followed by esterification of cinnamic acid with alcohol, yielding cinnamic acid esters.

**TABLE 3:** Scope of Pd_0.1_Cu_0.9_Co_2_O_4_NFs-catalyzed Heck coupling with concomitant transesterification reactions.

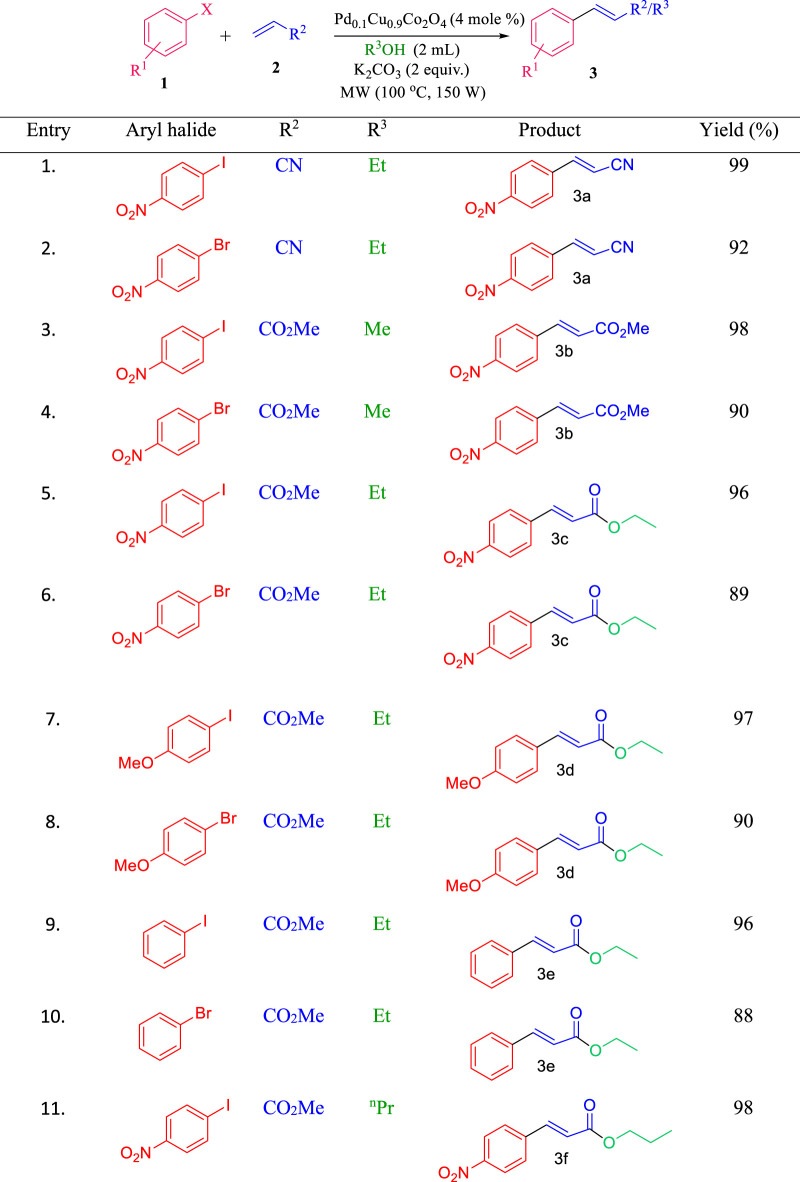 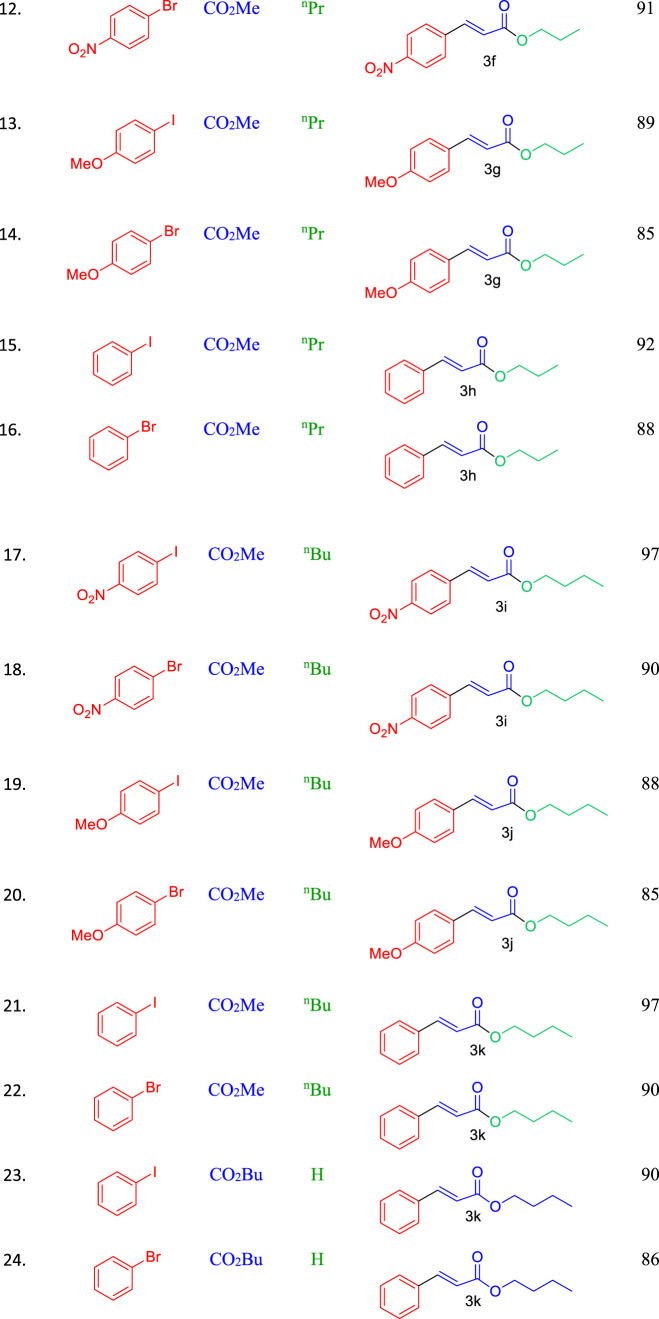 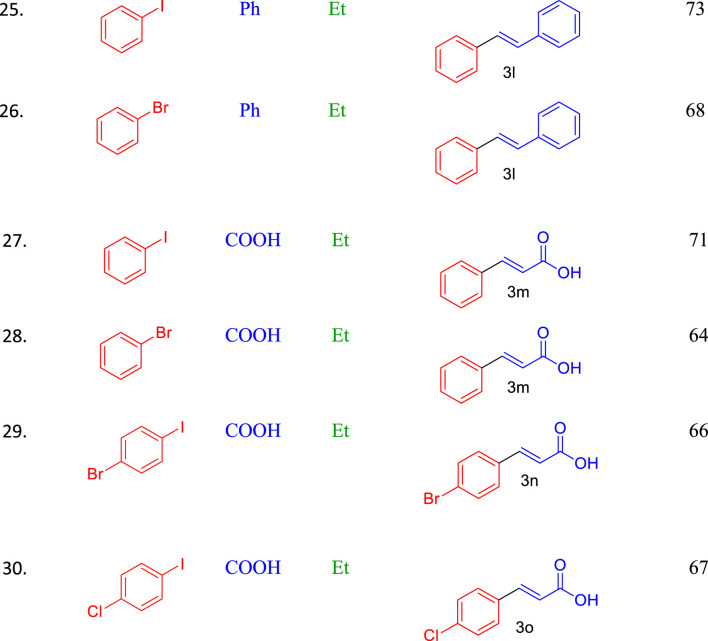

Conditions: aryl iodide (1.0 mmol), alkene (1.2 mmol), K_2_CO_3_ (2.0 equivalents), Pd_0.1_ Cu_0.9_Co_2_O_4_(4 mole %), alcohol solvent (2.0 mL), MW, 150 W, 80°C, 5 min.

**SCHEME 3:**
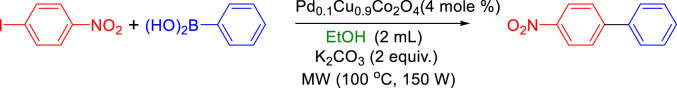
Synthesis of bi-aryl derivatives using Pd_0.1_Cu_0.9_Co_2_O_4_NFs catalyst via Suzuki coupling reactions.

We further assessed the catalytic performance of Pd₀.₁Cu₀.₉Co₂O₄ nano-flakes in another significant C–C bond-forming reaction: the Suzuki coupling of aryl halides with arylboronic acids to synthesize biaryl compounds. When a mixture of 1-nitro-4-iodobenzene (1 mmol), phenylboronic acid (1.2 mmol), and Pd₀.₁Cu₀.₉Co₂O₄ nano-flakes (10 mg) was heated at 100°C under microwave irradiation (150 W) in 2 mL of ethanol within a sealed microwave tube for 5 min, a quantitative yield of 4-nitrobiphenyl was obtained (Scheme 3).

**FIGURE 5:**
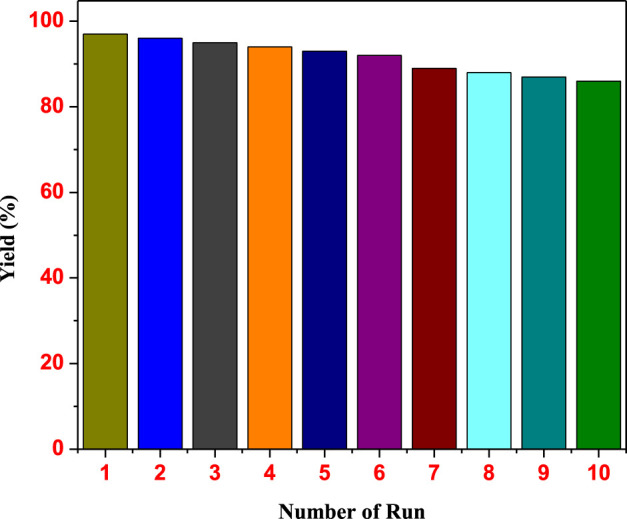
Recyclability of the Pd_0.1_Cu_0.9_Co_2_O_4_NFs for the Heck coupling reaction.

The scope of the Suzuki coupling reaction for synthesizing biaryl derivatives was explored using a straightforward and general experimental procedure, with the results summarized in Table 4. The Pd₀.₁Cu₀.₉Co₂O₄ nano-flakes efficiently catalyzed the coupling of aryl halides with aryl boronic acids under microwave irradiation, yielding various substituted biaryl derivatives. Arylboronic acids with a wide range of substituents produced robust yields of cross-coupled products. Notably, substrates bearing a–CO₂Me group (5o and 5q) also underwent transesterification. Additionally, sterically hindered boronic acids, such as 2,4,6-trisubstituted boronic acids, delivered high yields of biaryl products under the optimized reaction conditions (5k). The stability and reusability of the Pd_0.1_Cu_0.9_Co_2_O_4_ nano-flakes were evaluated using the Heck coupling of 1-iodo-4-nitrobenzene with acrylonitrile to form (E)-3-(4-nitrophenyl)acrylonitrile as a model reaction on a 2 mmol scale. After the reaction, the organic component was dissolved in ethyl acetate, and the catalyst was recovered by centrifugation. The recovered catalyst was washed, dried at 80°C for 4 h, and reused for ten consecutive runs. The recycling results, shown in Figure 5, indicate that the catalyst remained stable and active throughout the ten cycles, with no significant loss in efficiency or product yield. The slight decrease in yield could be attributed to catalyst loss during recycling or agglomeration of the nano- flakes during the process.

**TABLE 4:** Substrate scope of Pd_0.1_Cu_0.9_Co_2_O_4_NFs catalyzed Suzuki coupling of aryl halides and aryl boronic acids.

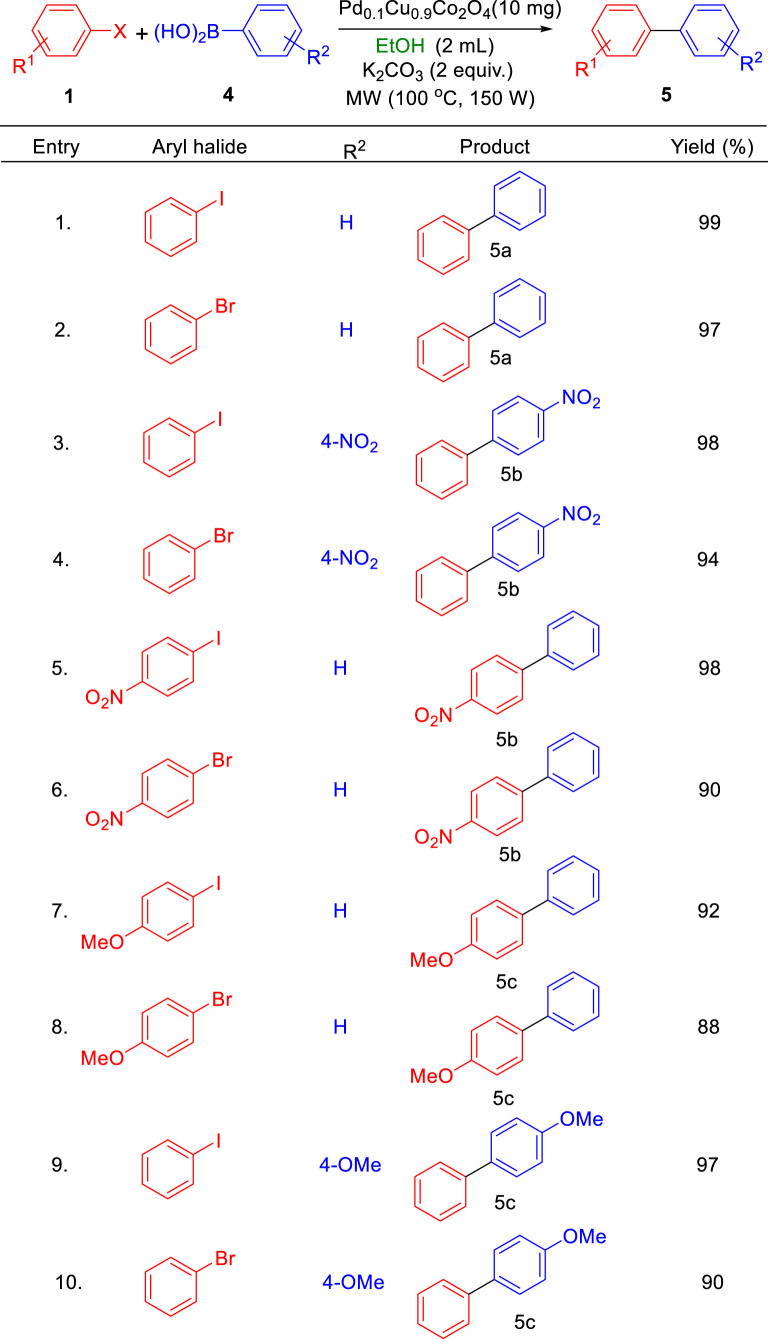 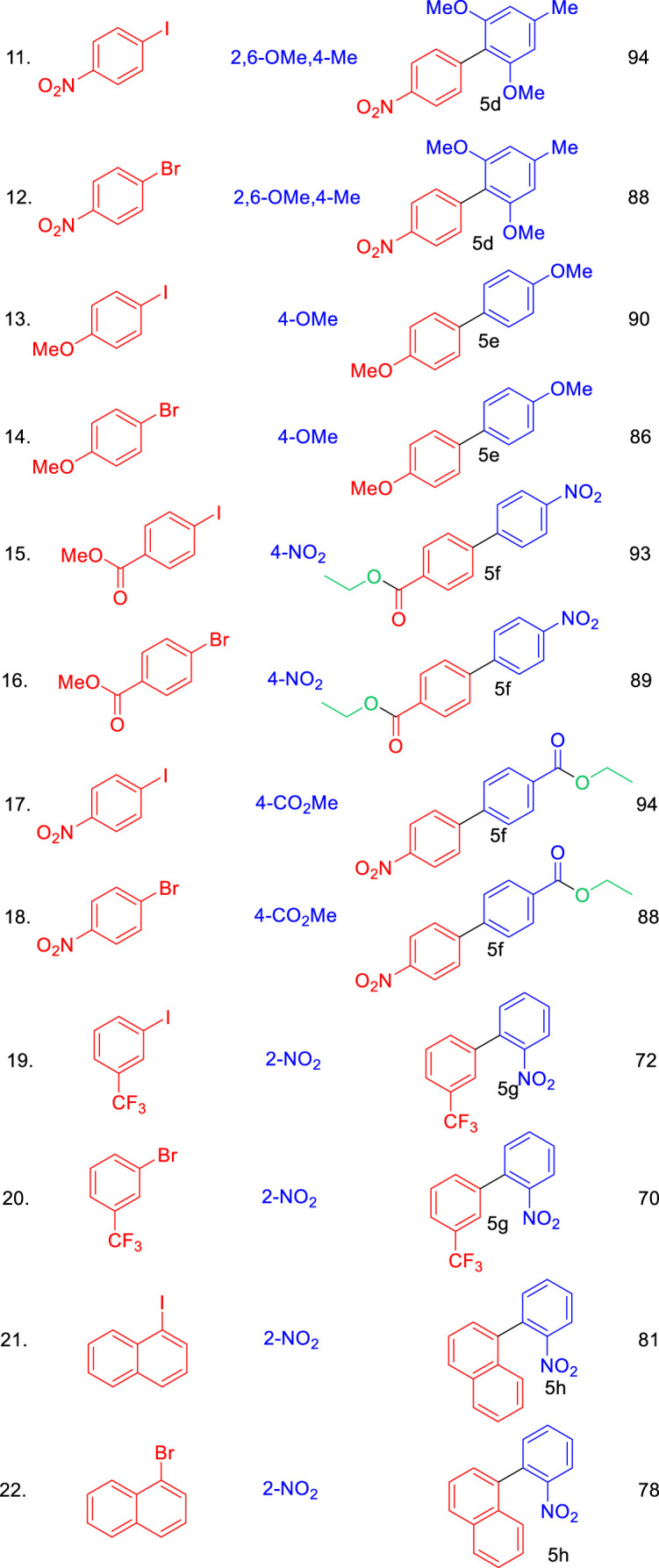 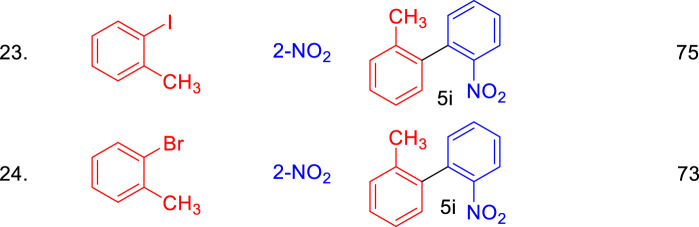

Conditions: aryl iodide (1.0 mmol), aryl boronic acid (1.2 mmol), K_2_CO_3_ (2.0 equi), Pd_0.1_Cu_0.9_Co_2_O_4_(10 mg), Ethanol (2.0 mL), MW, 150 W, 80°C, 5 min.

**FIGURE 6:**
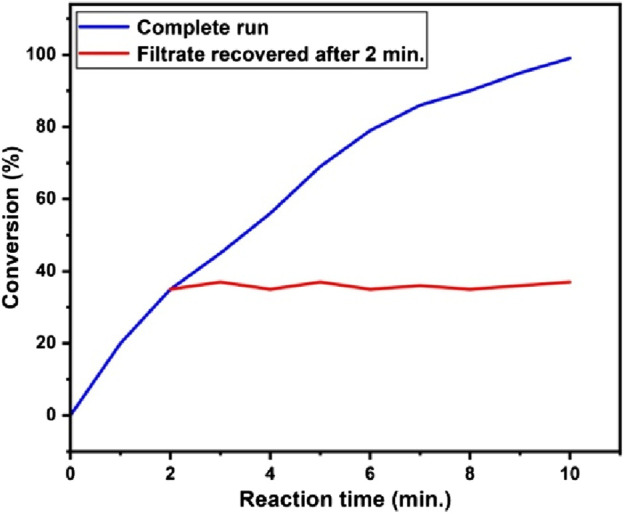
Results of the leaching study by hot-filtration test performed with **(A)** complete run (blue line), **(B)** filtrate removed after 2 min (red line) for the Heck-coupling cum transesterification reaction.

A hot filtration test was performed to assess the heterogeneity of the Pd_0.1_Cu_0.9_Co_2_O_4_ NFs catalyst through a leaching study. After 2 min of reaction (with 35% conversion achieved), the catalyst was removed from the reaction mixture using hot ultracentrifugation. The filtrate was then subjected to microwave (MW) irradiation for an additional 8 min, with reaction progress monitored at 2-min intervals. No further increase in product yield was observed after the catalyst was removed. As depicted in Figure 6, these results confirm that the Pd_0.1_Cu_0.9_Co_2_O_4_ NFs remained stable under the reaction conditions, with no detectable metal leaching from the catalyst.

**FIGURE 7:**
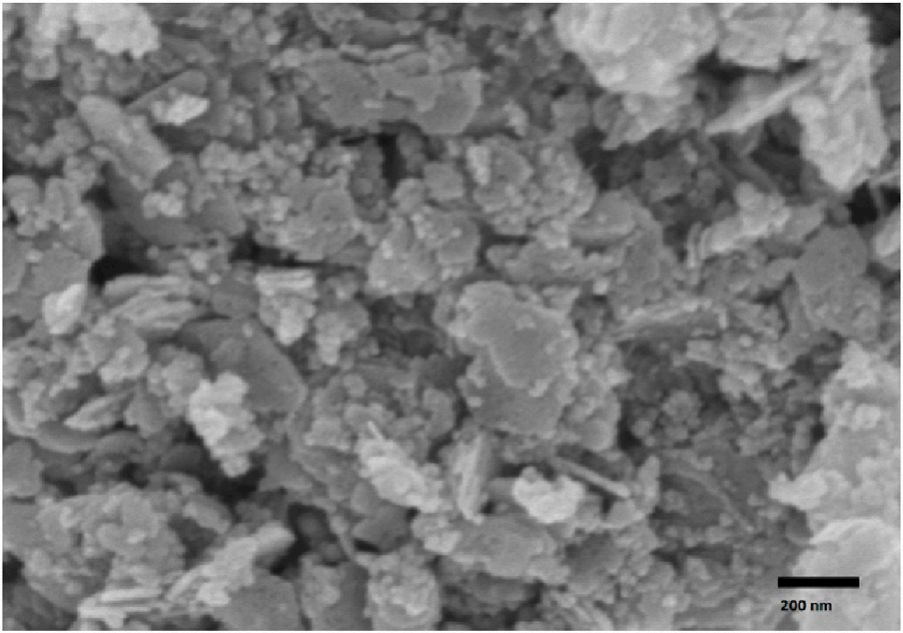
FESEM image of recycled catalyst after 10th run.

In the experiments, turnover number (TON) and turnover frequency (TOF) were determined using 10 mg of the Pd₀.₁Cu₀.₉Co₂O₄ catalyst, corresponding to a Pd content of 0.004 mol% in a 1 mmol scale reaction. For the Suzuki coupling reaction yielding biphenyl (5a), the calculated TON and TOF were 2500 and 15,000 h⁻^1^, respectively. Additionally, we conducted FESEM analysis to investigate the morphology of the reused catalyst. The FESEM image (Figure 7) of the catalyst after the 10th cycle confirmed that its flower-like structure remained intact, indicating stability and reusability of the catalyst.

The advantages of the Pd₀.₁Cu₀.₉Co₂O₄ nano-flakes catalyst for the Heck and Suzuki coupling reactions were highlighted by comparing it with previously reported Pd-based catalytic methods, as shown in Table 5. The comparison demonstrated that the Pd₀.₁Cu₀.₉Co₂O₄ catalyst outperforms other Pd-based spinel-structured catalysts, establishing it as a high-performance option in these reactions.

**TABLE 5:** Comparison of Present vs. Reported Methods for Cross-Coupling Reactions.

Sl. No.	Catalyst	Catalyst (mole%)<	Temp. (^o^C)	Time	Yield (%)	References
1	PdAl_2_O_4_	45	100	24 h	28–89	Kannan (2017)
2	Fe_3_O_4_-Pd-NHC	7.3	50	12 h	84–96	Stevens et al. (2005)
3	Pd/NiFe_2_O_4_	0.1	90	5–150 min	6–98	Borhade and Waghmode (2011)
4	PdCoFe_2_O_4_	3.2	Reflux	6–16 h	70–92	Senapati et al. (2012)
5	Fe_3_O_4_-DOPA-Pd	4.8	Ultra-sonication	1–5 min	45–90	Vaddula et al. (2012)
6	Pd-AcAc-Am-Fe_3_O_4_@Silica	0.28	80	1–3 h	80–98	Vibhute et al. (2020a)
7	Fe_3_O_4_@SiO_2_@mSiO_2_-Pd(II)	1.0	80	3–10	25–99.5	Le et al. (2014)
8	Fe_3_O_4_@SiO_2_@mSiO_2_–Pd (0)	0.075	80	6–8	56–97	Li et al. (2013)
9	Fe_3_O_4_@SiO_2_-Pd	0.03	85	20–100 min	85–96	Khazaei et al. (2017)
10	Pd-AcAc-Am-Fe_3_O_4_@SiO_2_	0.3	80	4 h	62–96	Vibhute et al. (2020b)
11	Pd-ZnFe_2_O_4_	9.24	reflux	2–12 h	85–94	Singh et al. (2013)
12	Pd_0.1_Cu_0.9_Co_2_O_4_	4	MW/150 W	10 min	86–99	This work

## Conclusion

In conclusion, we synthesized spinel-type Pd₀.₁Cu₀.₉Co₂O₄ nano-flakes via a simple co-precipitation method and characterized them using powder XRD, UV-Vis, FESEM, and EDX. The material showed excellent catalytic activity in Mizoroki-Heck and Suzuki cross-coupling reactions under microwave irradiation. Key advantages include the use of a mild base (K₂CO₃), ethanol as a green solvent, ligand-free conditions, short reaction times (10 min), and high yields (86%–99%). Notably, methyl acrylate underwent complete transesterification, while butyl acrylate yielded only cross-coupling products. The catalyst was stable and reusable for up to ten cycles with minimal loss in activity.

## Data Availability

The original contributions presented in the study are included in the article/Supplementary Material, further inquiries can be directed to the corresponding authors.
