# Supplementary material for: Hybrid Pd0.1Cu0.9Co2O4 nano-flakes: a novel, efficient and reusable catalyst for the one-pot heck and Suzuki couplings with simultaneous transesterification reactions under microwave irradiation
Source: Front Chem. 2024 Oct 30;12:1496234. doi: 10.3389/fchem.2024.1496234 (PMC11557397; doi:10.3389/fchem.2024.1496234)
Supplement: Supplementary file 1 [file DataSheet1.PDF]

SUPPORTING INFORMATION

Hybrid $Pd_{0.1}Cu_{0.9}Co_2O_4$ Nano-Flakes: A Novel, Efficient and Reusable Catalyst for the One-pot Heck and Suzuki Couplings with Simultaneous Transesterification Reactions under Microwave Irradiation

Ashok Raj Patel[a], Gurupada Maity[b], Tanmay K. Pati[c]*, Laksmikanta Adak[d], Christopher Cioffi[c], Subhash Banerjee[a]*

[a] Department of Chemistry, Guru Ghasidas Vishwavidyalaya, Koni, Bilaspur- 495009 (Chhattisgarh), India

[b] Department of Physics, School of Basic and Applied Science, Galgotias University, Gautam Buddh Nagar-203201, India

[c] Department of Chemistry, Rensselaer Polytechnic Institute, Troy, NY 12180, USA

[d] Department of Chemistry, Indian Institute of Engineering Science and Technology, Shibpur, Botanic Garden, Howrah 711103, India

* Correspondence: **Tanmay K. Pati** patit@rpi.edu, **Subhash Banerjee** ocsb2006@gmail.com

Keywords: Spinel-type catalyst, Nano-flake material, Mizoroki-Heck reaction, Suzuki coupling reaction, Transesterification, Microwave irradiation, Recyclable catalyst, Cross-coupling reactions

Sl. No.	Contents	Page No.
ESI 1	General information	**S2**
ESI 2	Method for the preparation of nano-$Pd_{0.1}$ $Cu_{0.9}Co_2O_4$	**S3**
ESI 3	General experimental procedure for nano-$Pd_{0.1}Cu_{0.9}Co_2O_4$ catalyzed Heck Coupling	**S3**
ESI 4	General experimental procedure for nano-$Pd_{0.1}Cu_{0.9}Co_2O_4$ catalyzed Suzuki Coupling	**S4**
ESI 5	Copies of ^{1}H NMR and ^{13}C NMR spectra of products listed in Table 03-04	**S5-42**
ESI 6	References	**S42**

ESI 1. General Information:

All chemicals and solvents were purchased from Merck, Sigma Aldrich, TCI-Chemicals, Sd-Fine, and HIMEDIA (India). Solvents were distilled before use, and all chemicals were used as received without further purification. Double-distilled water was employed for the preparation of carbon-supported nanoparticles and in organic reactions where required. Organic reactions were performed under ambient air, without the use of an inert atmosphere, and oven-dried glassware was used for all reactions. Thin-layer chromatography (TLC) was conducted on Merck 60F254 silica gel plates (0.25 mm thick), using ethyl acetate and petroleum ether as the eluting solvents.

The powder X-ray diffraction (XRD) analysis of nano-$Pd_{0.1}Cu_{0.9}Co_2O_4$ was carried out using a Rigaku Ultima IV X-ray diffractometer with Cu Kα radiation (λ = 1.540806 Å), operating at 40 kV and 40 mA, with a step size of 0.02° and a scanning rate of 0.24°/min. High-resolution transmission electron microscopy (HR-TEM) of nano-$Pd_{0.1}Cu_{0.9}Co_2O_4$ was performed using a JEOL 4000 EX/II operating at 400 kV (point-to-point resolution of 0.165 nm) and a JEOL 2010 FEG operating at 200 kV (information limit of 0.11 nm). Fourier-transform infrared (FT-IR) spectroscopy was conducted on a Bruker ALPHA-II using dichloromethane (DCM) as the solvent.

The ^1H and ^13C NMR spectra of the synthesized compounds were recorded using a Bruker spectrometer operating at 400 MHz and 101 MHz, respectively. Chemical shifts were referenced to the solvent peak in $CDCl_3$ (δ = 7.27 for ^1H and δ = 77 for ^13C). The NMR chemical shifts are reported as singlets (s), doublets (d), triplets (t), and multiplets (m), with coupling constants (J) expressed in hertz (Hz).

ESI 2. Method for the Preparation of nano-$Pd_{0.1}Cu_{0.9}Co_2O_4$:

$Pd_{0.1}Cu_{0.9}Co_2O_4$ was prepared via a straightforward co-precipitation method. Initially, 500 ml of double-distilled water was heated in a 1000 ml beaker, followed by the addition of 20.0 mmol (4.7586 g) of cobalt chloride ($CoCl_2·6H_2O$), which turned the solution crimson red. Subsequently, 9.0 mmol (1.5343 g) of copper chloride ($CuCl_2·2H_2O$) and 1.0 mmol (177.33 mg) of palladium chloride ($PdCl_2$) (dissolved in 50% HCl) were added, changing the solution color to pink and then wine red.

A sodium hydroxide (NaOH) solution was prepared in double-distilled water and added dropwise to the resulting solution with continuous stirring until the pH reached 10.0. A blue precipitate formed, and the solution gradually turned grey. The mixture was stirred for 2 hours, after which the solution was repeatedly washed with distilled water to remove residual alkali, adjusting the pH to 6.0-7.0. The precipitate was filtered using Whatman filter paper no. 42 and dried in a hot air oven overnight at 80°C.

Finally, the solid material was ground and calcined at 350°C for 3 hours. The resulting black material was characterized using various spectroscopic and analytical techniques.

ESI 3. General experimental procedure for nano-$Pd_{0.1}$ $Cu_{0.9}Co_2O_4$ catalyzed Heck Coupling:

Representative procedure for the coupling of 1-iodo-4-nitrobenzene and acrylonitrile (3a, Table 3): : A mixture of 1-iodo-4-nitrobenzene (249 mg, 1.0 mmol), acrylonitrile (64 mg, 1.2 mmol), K_2CO_3 (276 mg, 2.0 mmol), nano-$Pd_{0.1}Cu_{0.9}Co_2O_4$ (4 mole %), and ethanol (2 mL) was added to a sealed microwave (MW) tube (10 mL). The reaction was irradiated under MW conditions at 150 W for 10 minutes, with the progress monitored by thin-layer chromatography (TLC). Upon completion, the reaction mixture was cooled to room temperature, and the catalyst (nano-$Pd_{0.1}Cu_{0.9}Co_2O_4$) was separated by filtration and washed with 5 mL of ethyl acetate. The excess acrylonitrile and solvents from the combined filtrate were removed under reduced pressure using a rotary evaporator, yielding a crude solid. This solid was washed with distilled water and extracted with ethyl acetate to afford pure (E)-3-(4-nitrophenyl)acrylonitrile (**3a**) as a white to

yellowish solid (174 mg, 100% yield). The product was characterized by its melting point and ^1H and ^{13}C NMR spectroscopy.

Melting point observed: 192-195 °C; reported 203-204 °C; **^{1}H NMR (400 MHz, $CDCl_3$) δ =** 8.28(d, *J*=8.0 Hz, 2H), 7.63(d, *J* = 8.0 Hz, 2H), 7.48(d, *J* = 16.0 Hz, 1H), 6.05(d, *J* = 16.0 Hz, 1H) ppm; **^{13}C NMR (101 MHz, $CDCl_3$) δ =** 149.0, 147.7, 139.1**,** 128.1, 124.4, 116.9, 101.0 ppm. These data are in well accordance with those of reported one.[1]

A similar experimental procedure was followed to obtain pure products listed in Table 03.

ESI 4. General experimental procedure for nano-$Pd_{0.1}$ $Cu_{0.9}Co_2O_4$ catalyzed Suzuki Coupling:

***Representative procedure for the coupling of 1-iodo-4- nitrobenzene and phenyl boronic acid (5a,** Table 4):* A mixture of 1-iodo-4-nitrobenzene (249 mg, 1.0 mmol), phenylboronic acid (146 mg, 1.2 mmol), K_2CO_3 (276 mg, 2.0 mmol), nano-$Pd_{0.1}Cu_{0.9}Co_2O_4$ (10 mg), and ethanol (2 mL) was placed in a sealed microwave (MW) tube (10 mL) and irradiated at 150 W for 10 minutes. The reaction progress was monitored by thin-layer chromatography (TLC). Upon completion, the mixture was cooled to room temperature, and the catalyst (nano-$Pd_{0.1}Cu_{0.9}Co_2O_4$) was separated by filtration and washed with 5 mL of ethyl acetate. The solvent from the combined filtrate was removed under reduced pressure using a rotary evaporator. The resulting solid was washed with distilled water to remove any excess phenylboronic acid and extracted with ethyl acetate to yield pure 4-nitrobiphenyl as a yellow solid (199 mg, 100% yield). The identity of the product was confirmed by its melting point and ^1H and ^13C NMR spectroscopic analyses. Melting point observed: 112-114 °C; reported 113-114 °C; ^{1}H NMR (500 MHz, $CDCl_3$) δ = 8.2(d, *J*=8.0 Hz, 2H), 7.7(d, *J* = 8.0 Hz, 2H), 7.6(d, *J* = 8.0 Hz, 2H), 7.5-7.4(m, 3H) ppm; ^{13}C NMR (125 MHz, $CDCl_3$) δ = 147.6, 147.1, 138.7, 129.1, 128.9, 127.8, 127.4, 124.1 ppm . These data are in well accordance with those of reported one.[2]

A similar experimental procedure was followed for all the reactions listed in Table 4. However, for liquid products the product was extracted with ethyl acetate, washed with water, dried over Na2SO4, filtered and organic solvent was evaporated under reduced pressure to provide the crude product. The pure products were obtained by column chromatography over silica gel (60–120 mesh) using mixture of petroleum ether and ethyl acetate (90:10) as an eluting solvent to afford the pure product.

ESI 5. Copies of ^{1}H NMR and ^{13}C NMR spectra of products listed in Table 3-4:

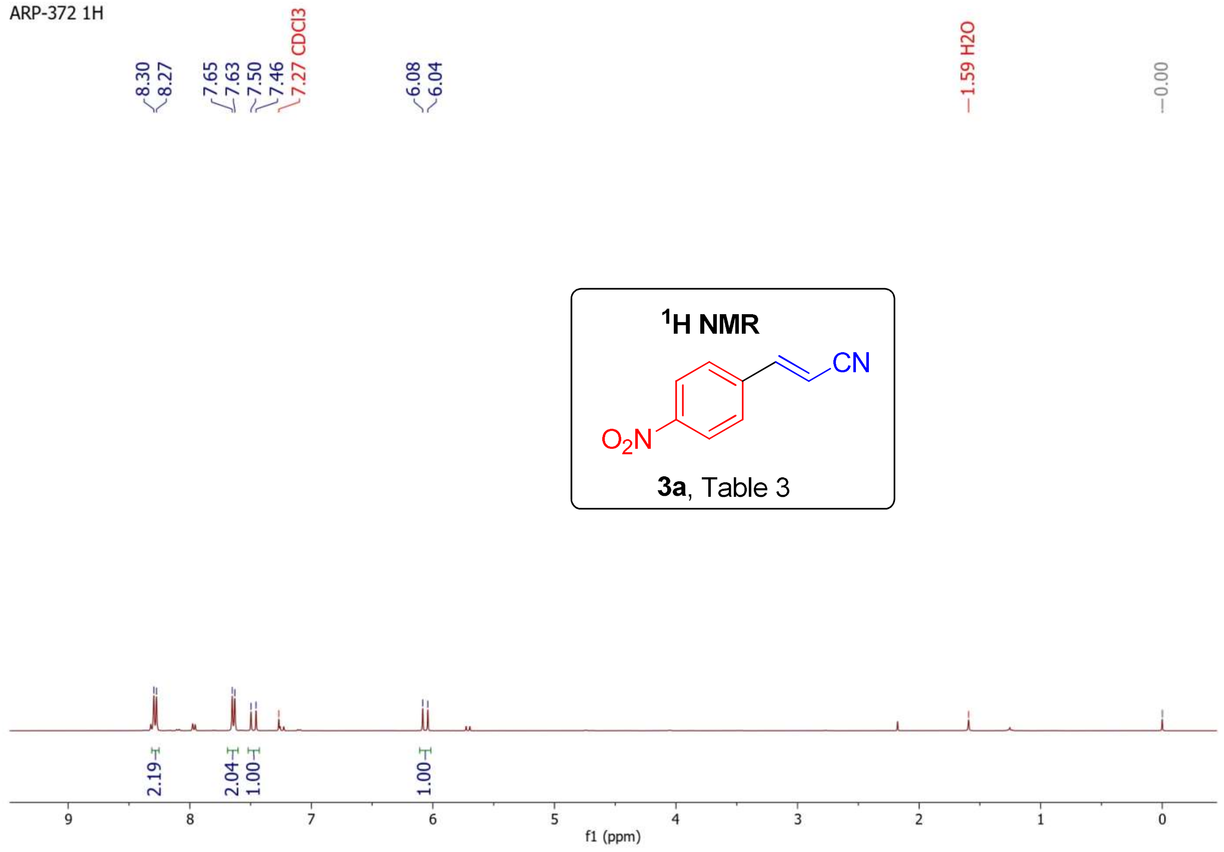

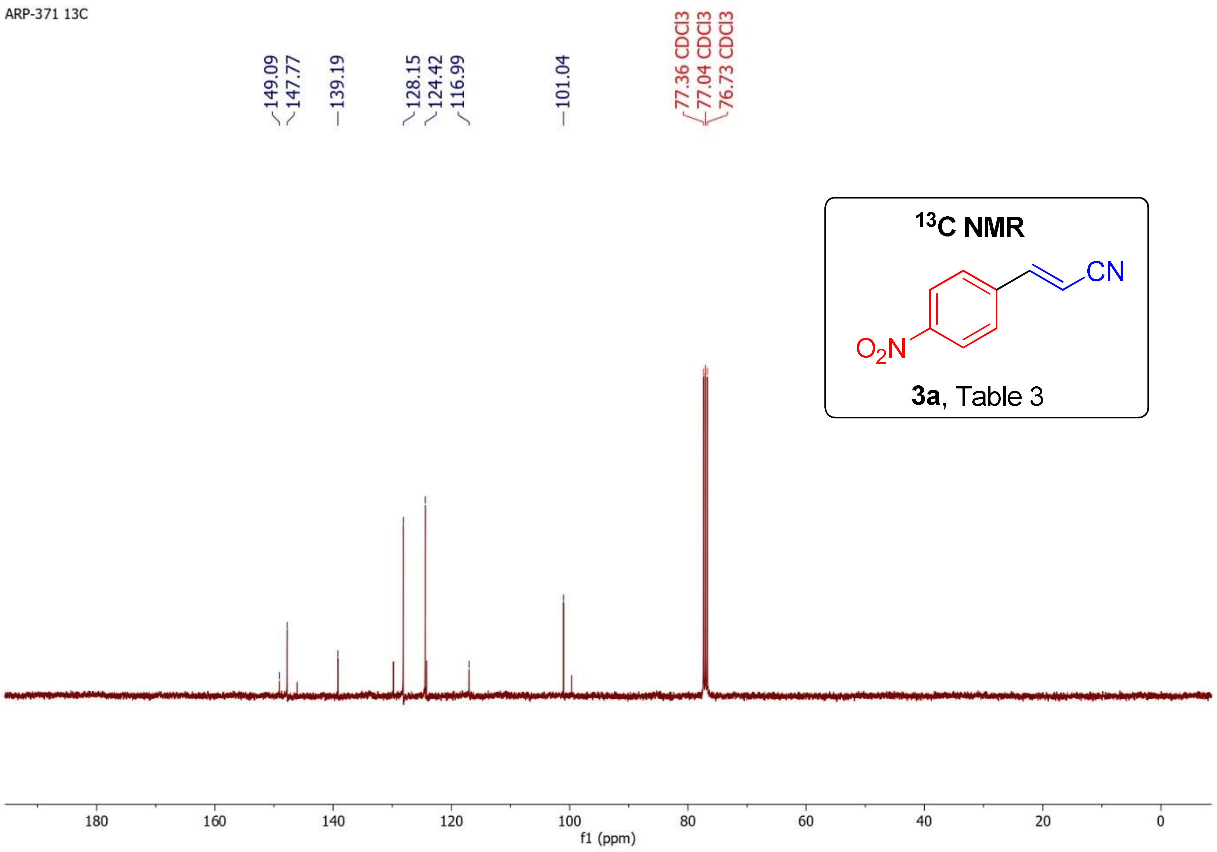
ARP-371 13C
149.09
147.77
139.19
128.15
124.42
116.99
101.04
77.36 CDCl3
77.04 CDCl3
76.73 CDCl3
13C NMR
CN
O2N
3a, Table 3
180
160
140
120
100
80
60
40
20
0
f1 (ppm)

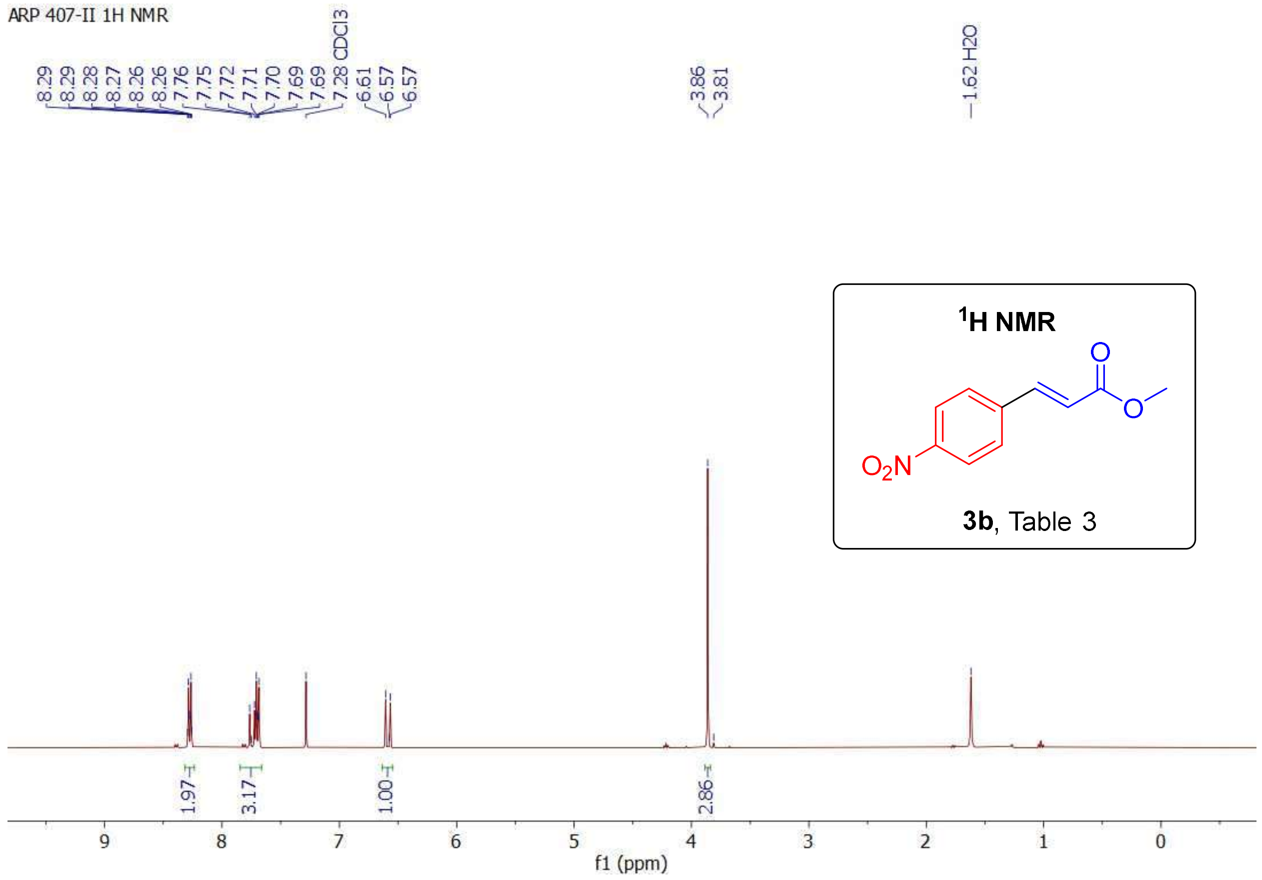
ARP 407-II 1H NMR
8.29
8.29
8.28
8.27
8.26
8.26
7.76
7.75
7.72
7.71
7.70
7.69
7.69
7.28 CDCl3
6.61
6.57
6.57
3.86
3.81
1.62 H2O
1H NMR
O2N
3b, Table 3
1.97
3.17
1.00
2.86
9
8
7
6
5
4
3
2
1
0
f1 (ppm)

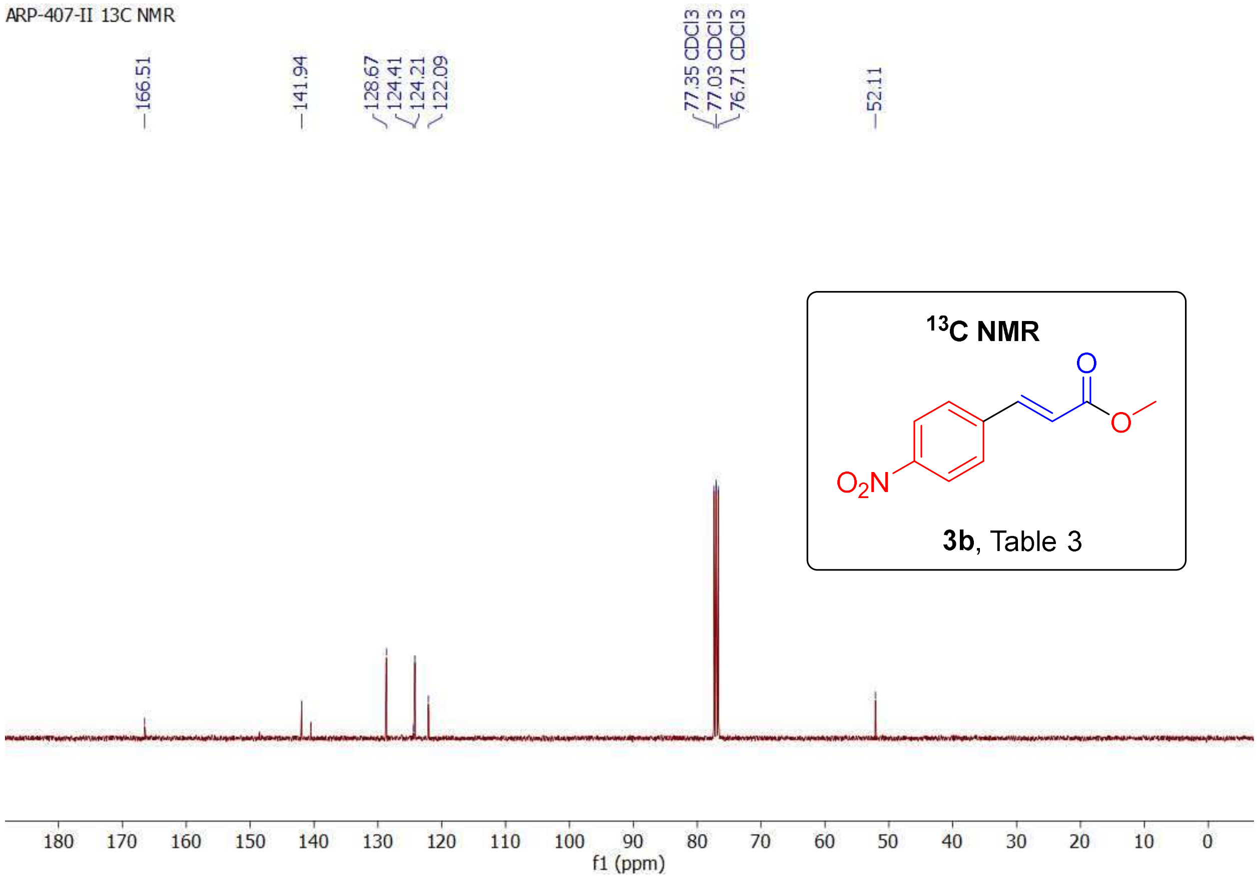
ARP-407-II 13C NMR
166.51
141.94
128.67
124.41
124.21
122.09
77.35 CDCl3
77.03 CDCl3
76.71 CDCl3
52.11
13C NMR
O
O
O2N
3b, Table 3
180 170 160 150 140 130 120 110 100 90 80 70 60 50 40 30 20 10 0
f1 (ppm)

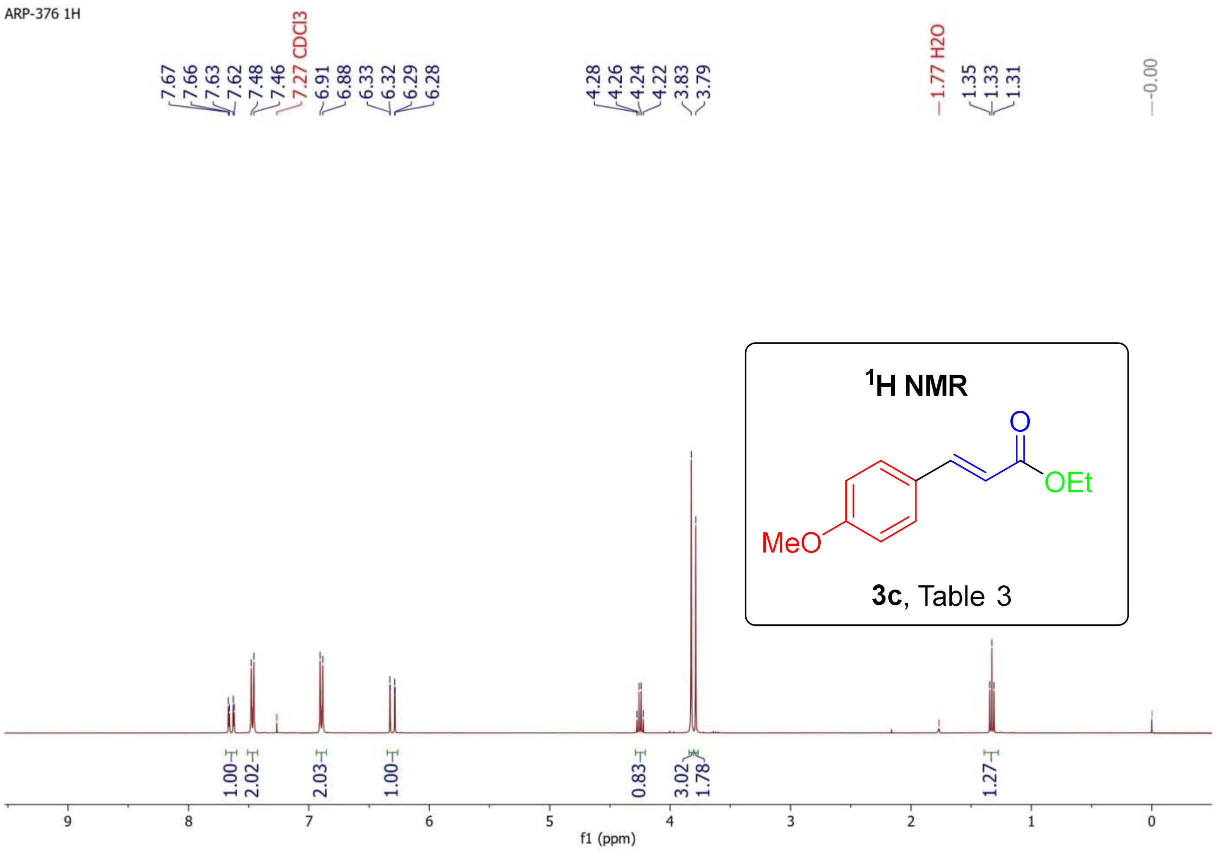
ARP-376 1H
7.67
7.66
7.63
7.62
7.48
7.46
7.27 CDCl3
6.91
6.88
6.33
6.32
6.29
6.28
4.28
4.26
4.24
4.22
3.83
3.79
1.77 H2O
1.35
1.33
1.31
0.00
1H NMR
O
OEt
MeO
3c, Table 3
1.00
2.02
2.03
1.00
0.83
3.02
1.78
1.27
9
8
7
6
5
4
3
2
1
0
f1 (ppm)

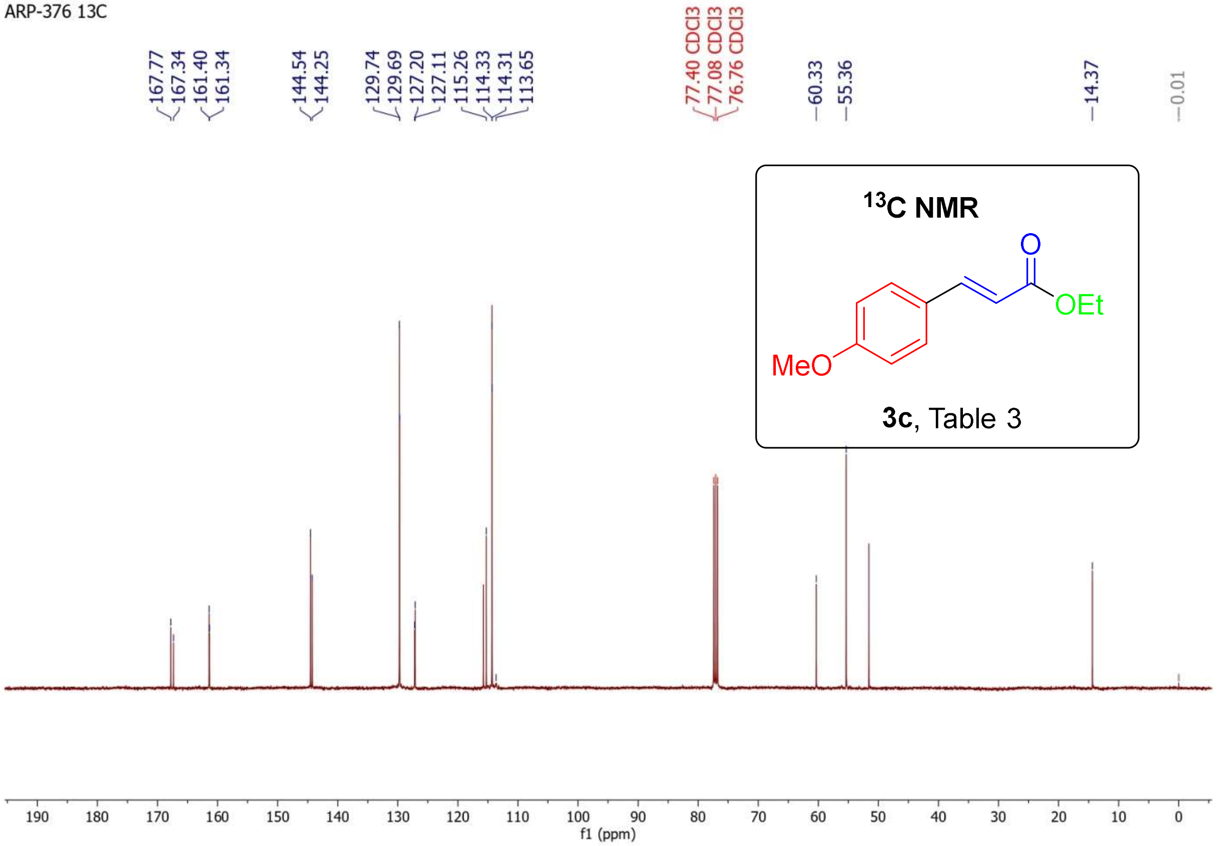
ARP-376 13C
167.77
167.34
161.40
161.34
144.54
144.25
129.74
129.69
127.20
127.11
115.26
114.33
114.31
113.65
77.40 CDCl3
77.08 CDCl3
76.76 CDCl3
60.33
55.36
14.37
0.01
13C NMR
O
OEt
MeO
3c, Table 3
190 180 170 160 150 140 130 120 110 100 90 80 70 60 50 40 30 20 10 0
f1 (ppm)

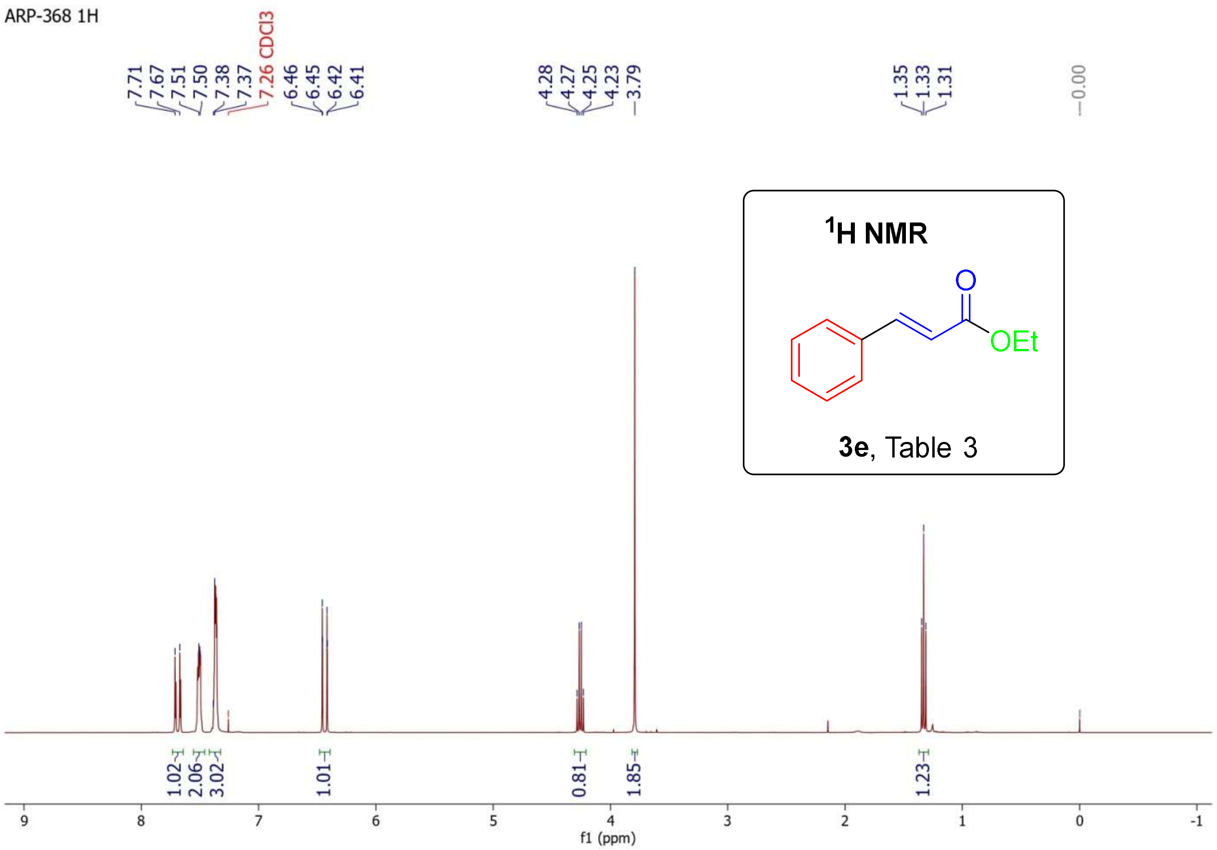

ARP-368 1H
7.71
7.67
7.51
7.50
7.38
7.37
7.26 CDCl3
6.46
6.45
6.42
6.41
4.28
4.27
4.25
4.23
3.79
1.35
1.33
1.31
0.00
1H NMR
O
OEt
3e, Table 3
1.02
2.06
3.02
1.01
0.81
1.85
1.23
9
8
7
6
5
4
3
2
1
0
-1
f1 (ppm)

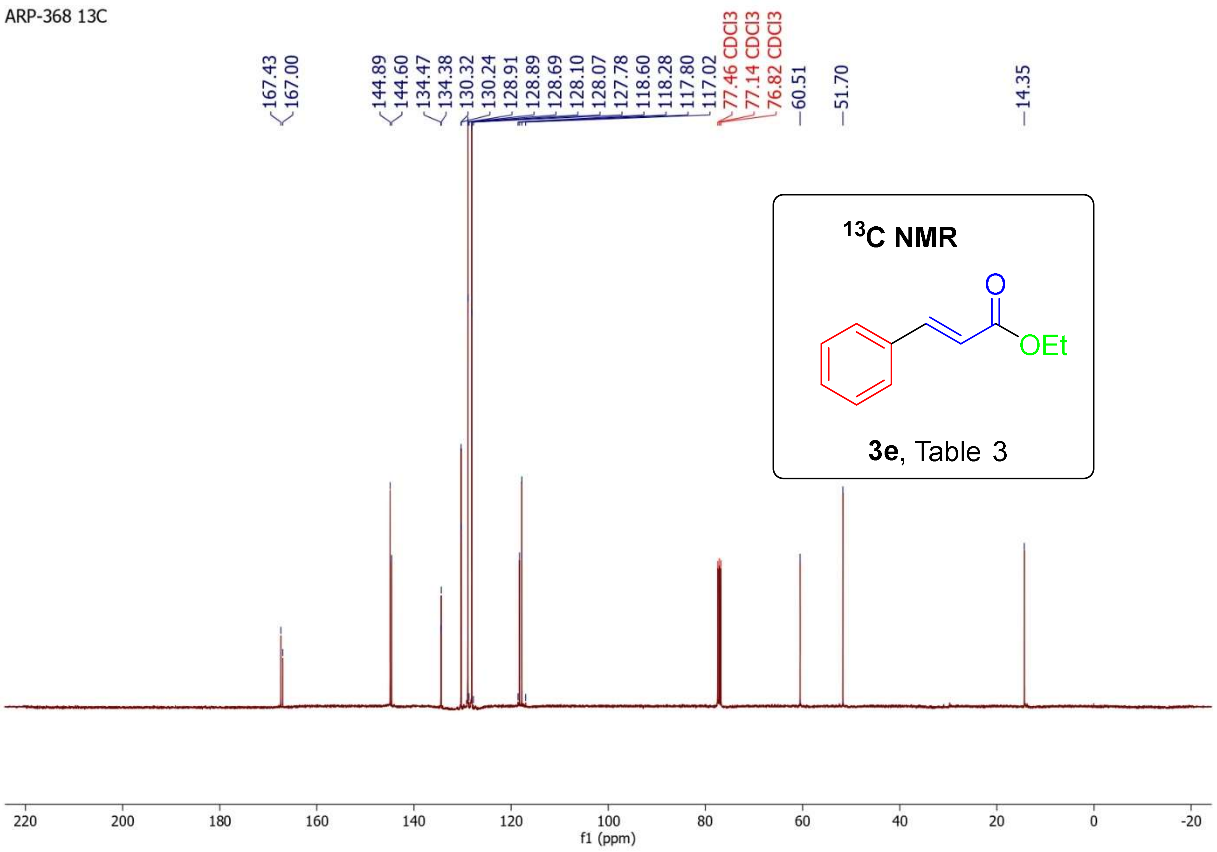
ARP-368 13C
167.43
167.00
144.89
144.60
134.47
134.38
130.32
130.24
128.91
128.89
128.69
128.10
128.07
127.78
118.60
118.28
117.80
117.02
77.46 CDCl3
77.14 CDCl3
76.82 CDCl3
60.51
51.70
14.35
13C NMR
O
OEt
3e, Table 3
220
200
180
160
140
120
100
80
60
40
20
0
-20
f1 (ppm)

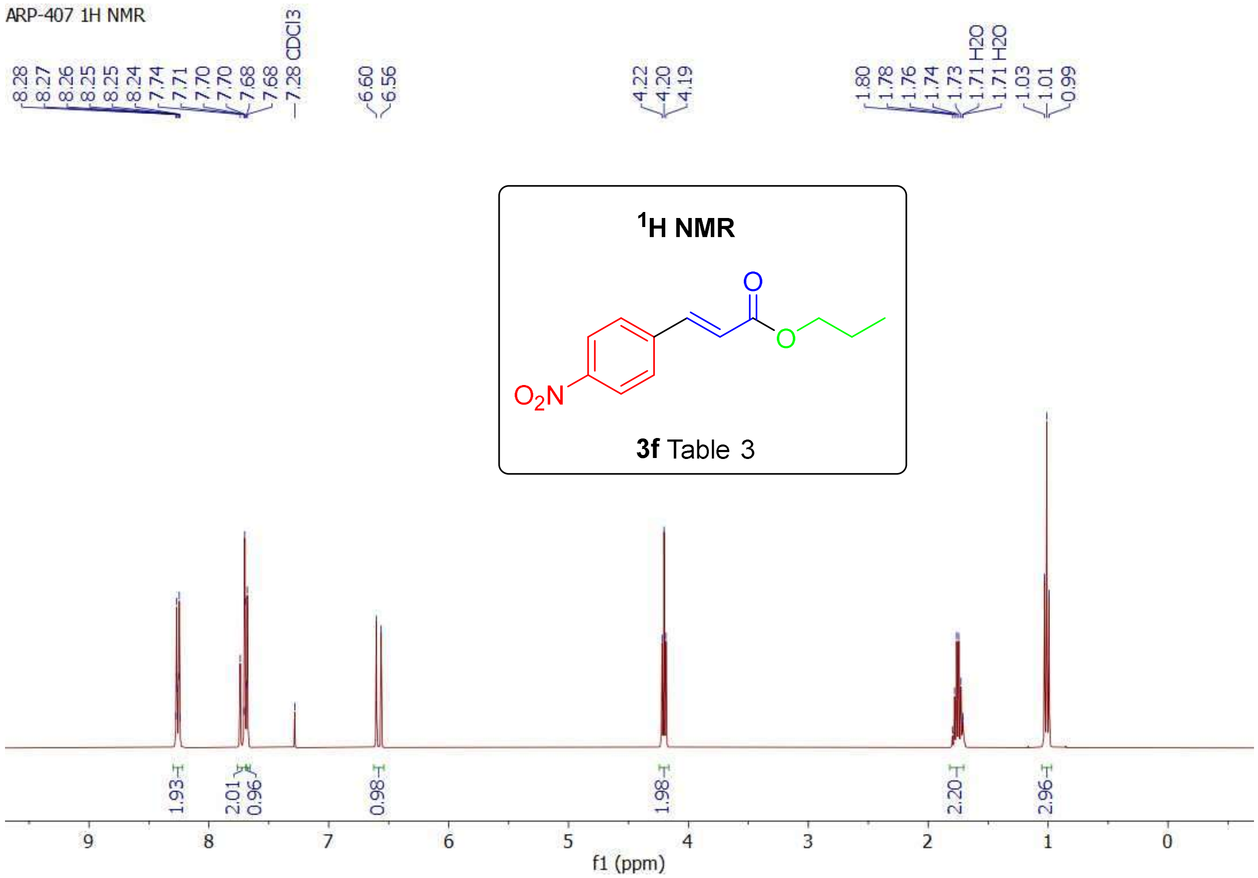
ARP-407 1H NMR
8.28
8.27
8.26
8.25
8.25
8.24
7.74
7.71
7.70
7.70
7.68
7.68
7.28 CDCl3
6.60
6.56
4.22
4.20
4.19
1.80
1.78
1.76
1.74
1.73
1.71 H2O
1.71 H2O
1.03
1.01
0.99
1H NMR
O
O
O2N
3f Table 3
1.93
2.01
0.96
0.98
1.98
2.20
2.96
9
8
7
6
5
4
3
2
1
0
f1 (ppm)

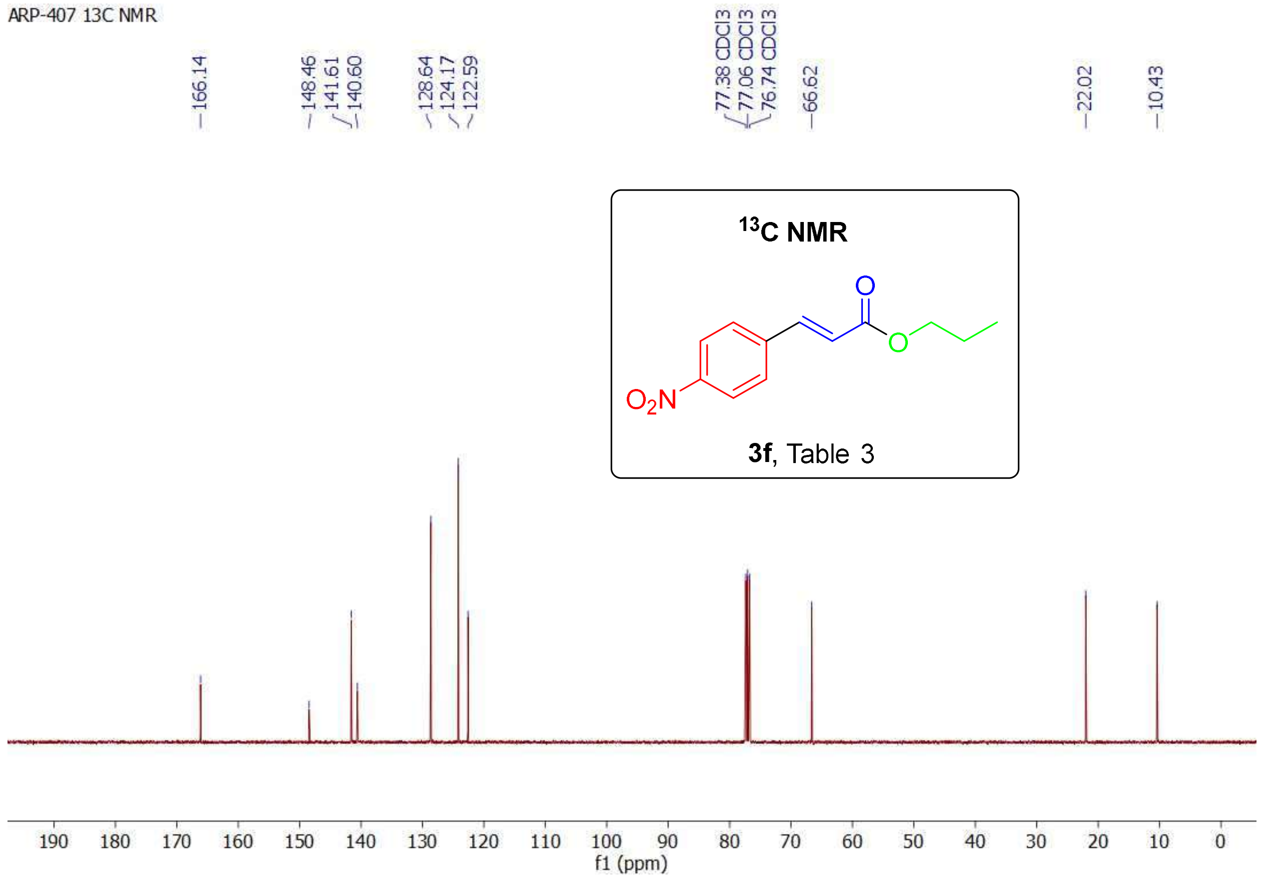

ARP-407 13C NMR
166.14
148.46
141.61
140.60
128.64
124.17
122.59
77.38 CDCl3
77.06 CDCl3
76.74 CDCl3
66.62
22.02
10.43
13C NMR
O
O
O2N
3f, Table 3
190 180 170 160 150 140 130 120 110 100 90 80 70 60 50 40 30 20 10 0
f1 (ppm)

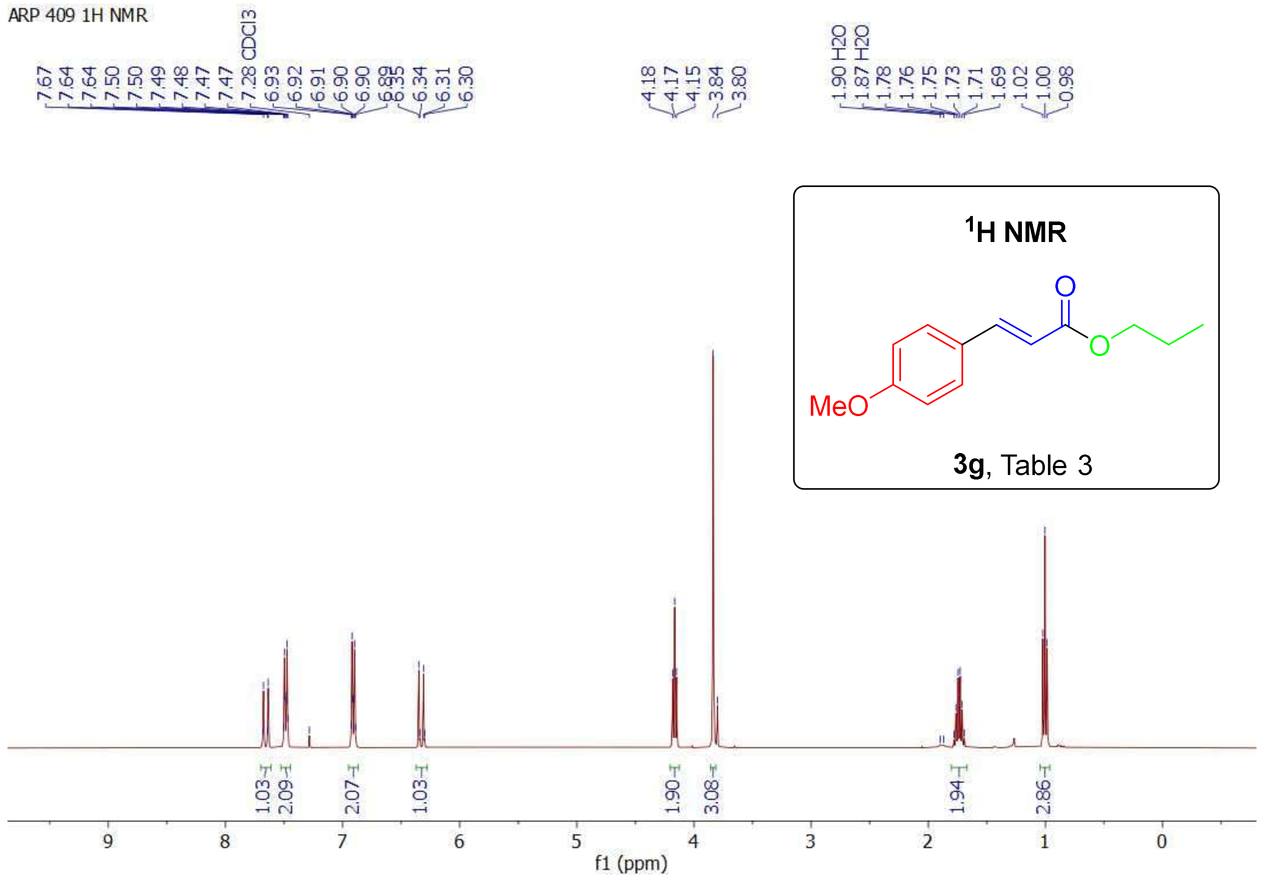

ARP 409 1H NMR
7.67
7.64
7.64
7.50
7.50
7.49
7.48
7.47
7.47
7.28 CDCl3
6.93
6.92
6.91
6.90
6.90
6.89
6.35
6.34
6.31
6.30
4.18
4.17
4.15
3.84
3.80
1.90 H2O
1.87 H2O
1.78
1.76
1.75
1.73
1.71
1.69
1.02
1.00
0.98
1H NMR
MeO
O
O
3g, Table 3
1.03
2.09
2.07
1.03
1.90
3.08
1.94
2.86
9
8
7
6
5
4
3
2
1
0
f1 (ppm)

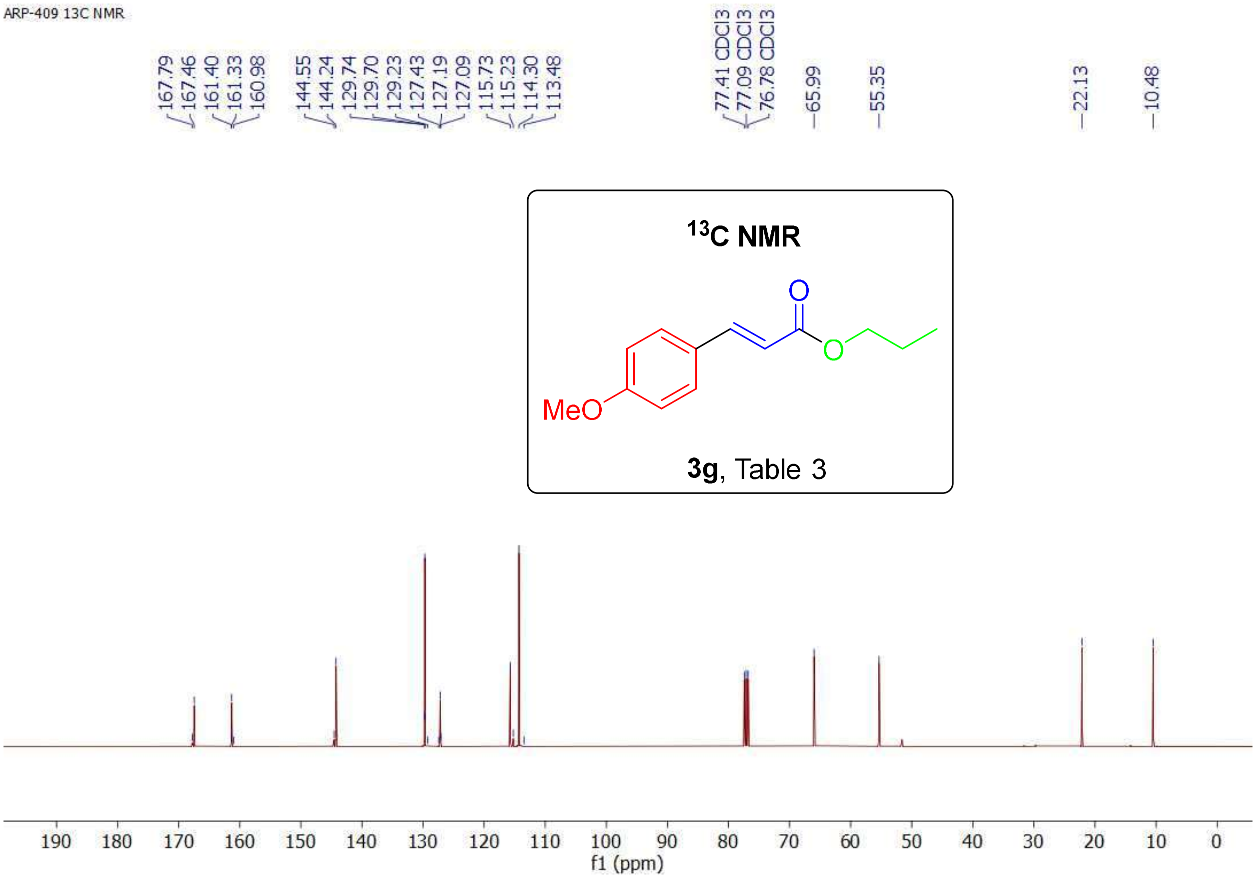
ARP-409 13C NMR
167.79
167.46
161.40
161.33
160.98
144.55
144.24
129.74
129.70
129.23
127.43
127.19
127.09
115.73
115.23
114.30
113.48
77.41 CDCl3
77.09 CDCl3
76.78 CDCl3
65.99
55.35
22.13
10.48
13C NMR
MeO
O
O
3g, Table 3
190 180 170 160 150 140 130 120 110 100 90 80 70 60 50 40 30 20 10 0
f1 (ppm)

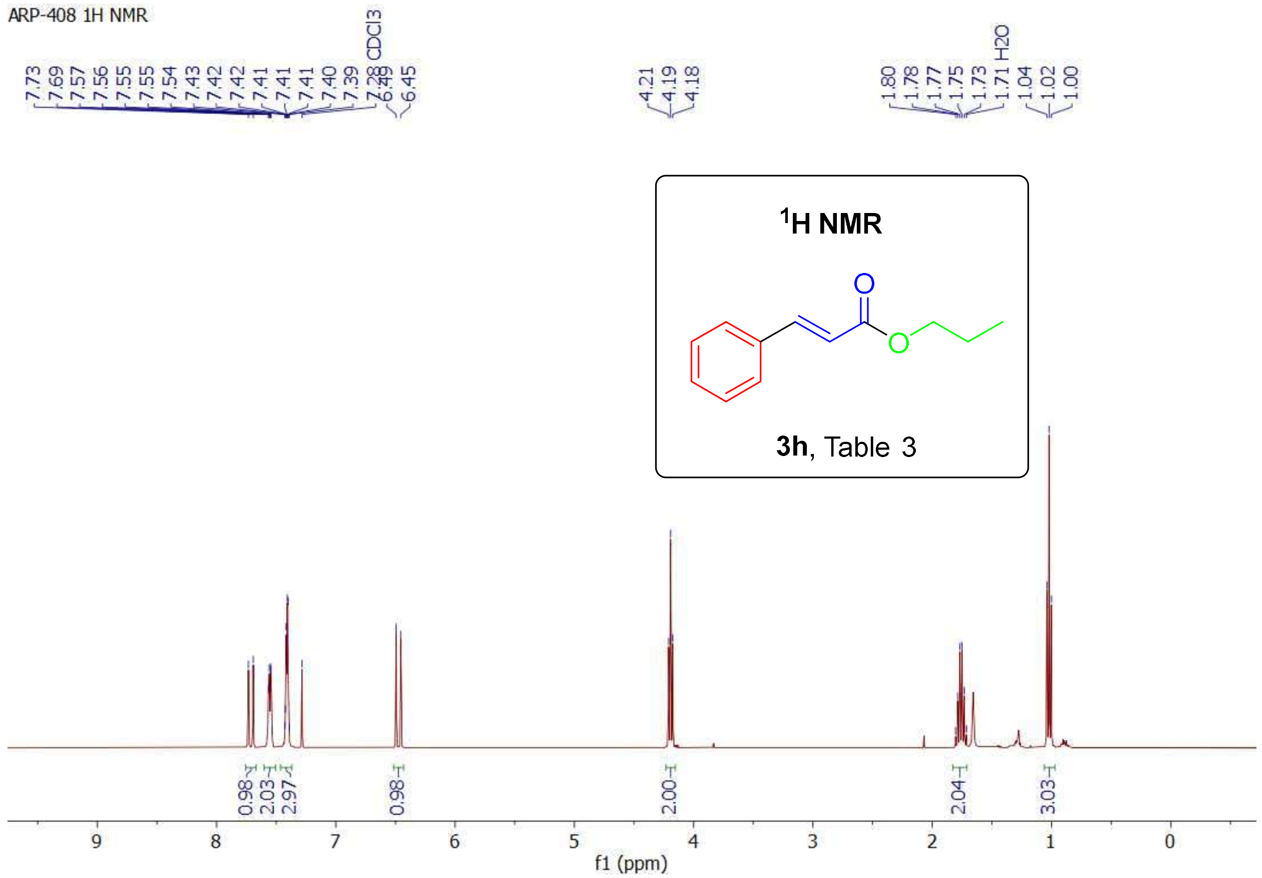

ARP-408 1H NMR
7.73
7.69
7.57
7.56
7.55
7.55
7.54
7.43
7.42
7.42
7.41
7.41
7.41
7.40
7.39
7.28 CDCl3
6.49
6.45
4.21
4.19
4.18
1.80
1.78
1.77
1.75
1.73
1.71 H2O
1.04
1.02
1.00
1H NMR
3h, Table 3
0.98
2.03
2.97
0.98
2.00
2.04
3.03
f1 (ppm)

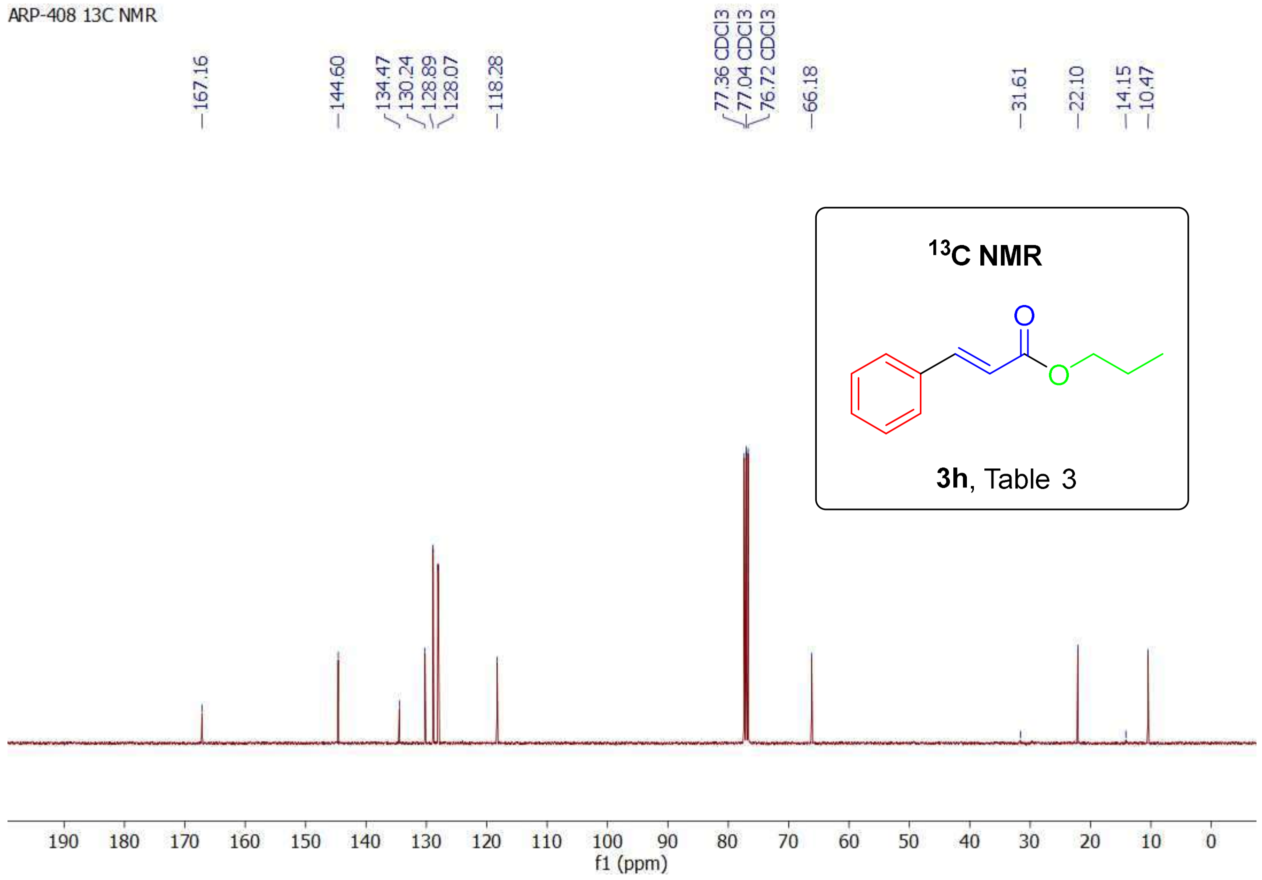
ARP-408 13C NMR
167.16
144.60
134.47
130.24
128.89
128.07
118.28
77.36 CDCl3
77.04 CDCl3
76.72 CDCl3
66.18
31.61
22.10
14.15
10.47
^{13}C NMR
3h, Table 3
190 180 170 160 150 140 130 120 110 100 90 80 70 60 50 40 30 20 10 0
f1 (ppm)

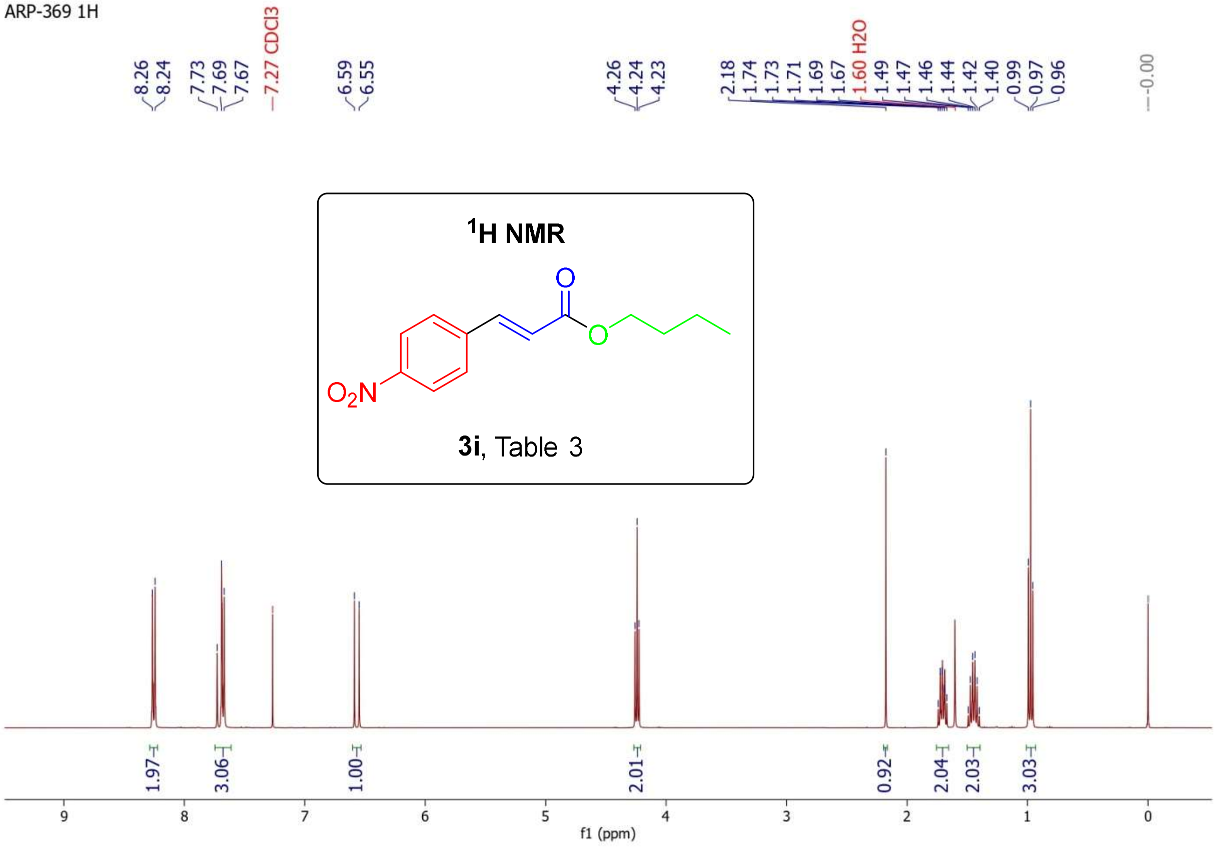
ARP-369 1H
8.26
8.24
7.73
7.69
7.67
7.27 CDCl3
6.59
6.55
4.26
4.24
4.23
2.18
1.74
1.73
1.71
1.69
1.67
1.60 H2O
1.49
1.47
1.46
1.44
1.42
1.40
0.99
0.97
0.96
0.00
1H NMR
O
O
O2N
3i, Table 3
1.97
3.06
1.00
2.01
0.92
2.04
2.03
3.03
9
8
7
6
5
4
3
2
1
0
f1 (ppm)

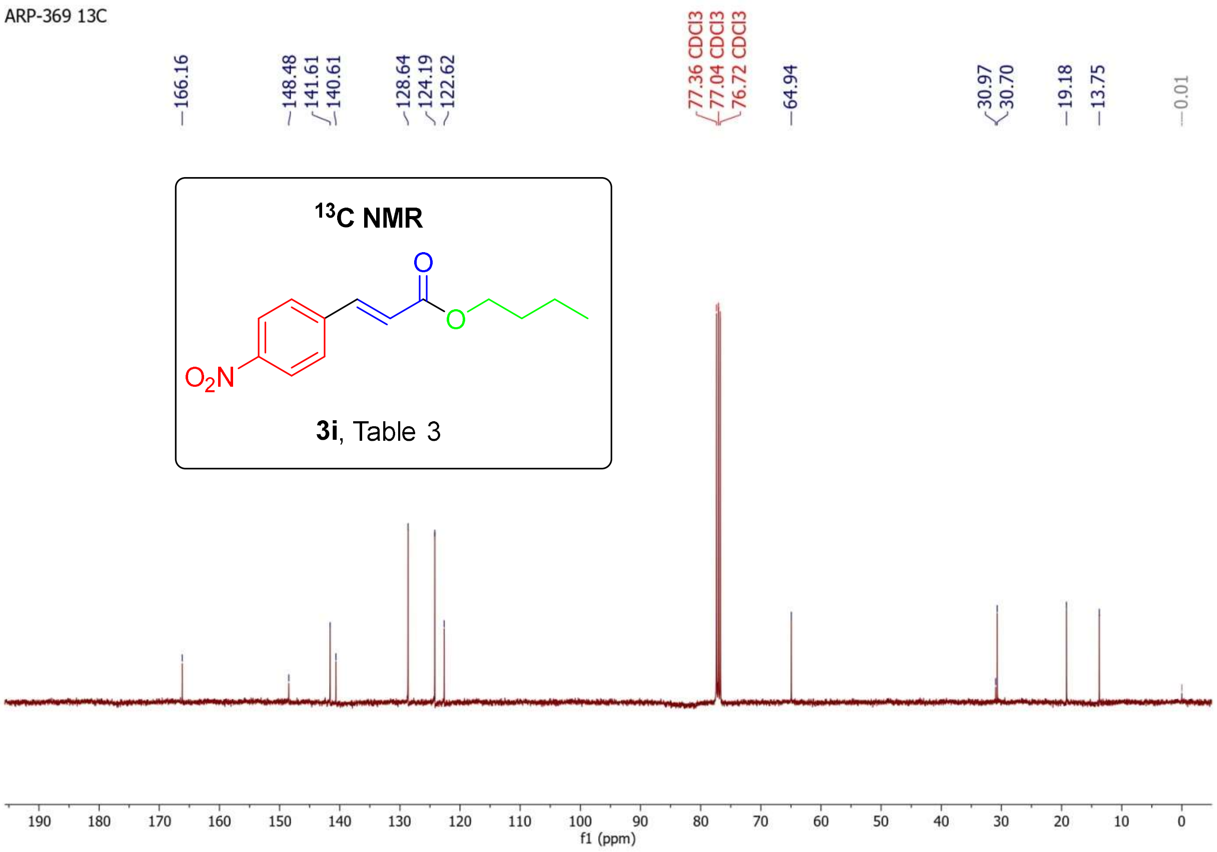
ARP-369 13C
166.16
148.48
141.61
140.61
128.64
124.19
122.62
77.36 CDCl3
77.04 CDCl3
76.72 CDCl3
64.94
30.97
30.70
19.18
13.75
-0.01
13C NMR
O
O
O2N
3i, Table 3
190 180 170 160 150 140 130 120 110 100 90 80 70 60 50 40 30 20 10 0
f1 (ppm)

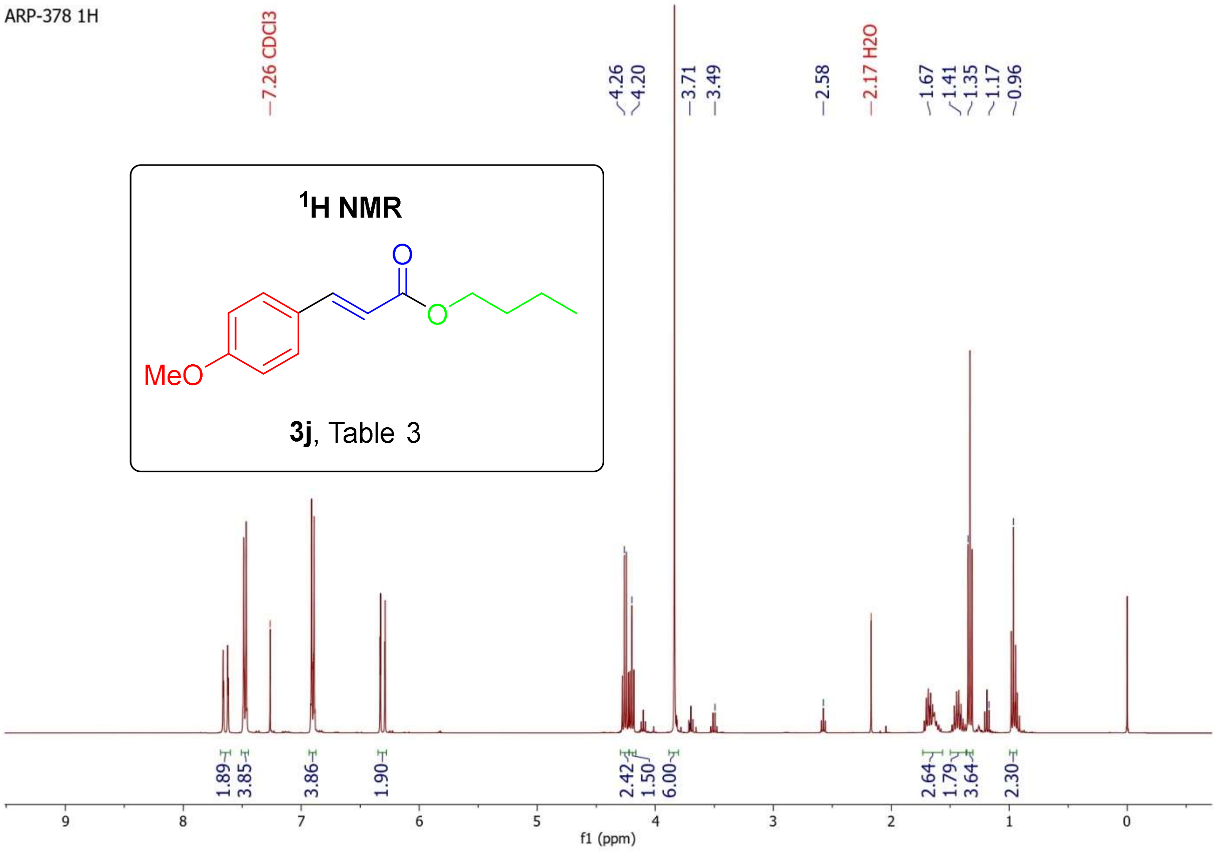

ARP-378 1H
7.26 CDCl3
4.26
4.20
3.71
3.49
2.58
2.17 H2O
1.67
1.41
1.35
1.17
0.96
1H NMR
MeO
O
O
3j, Table 3
1.89
3.85
3.86
1.90
2.42
1.50
6.00
2.64
1.79
3.64
2.30
9
8
7
6
5
4
3
2
1
0
f1 (ppm)

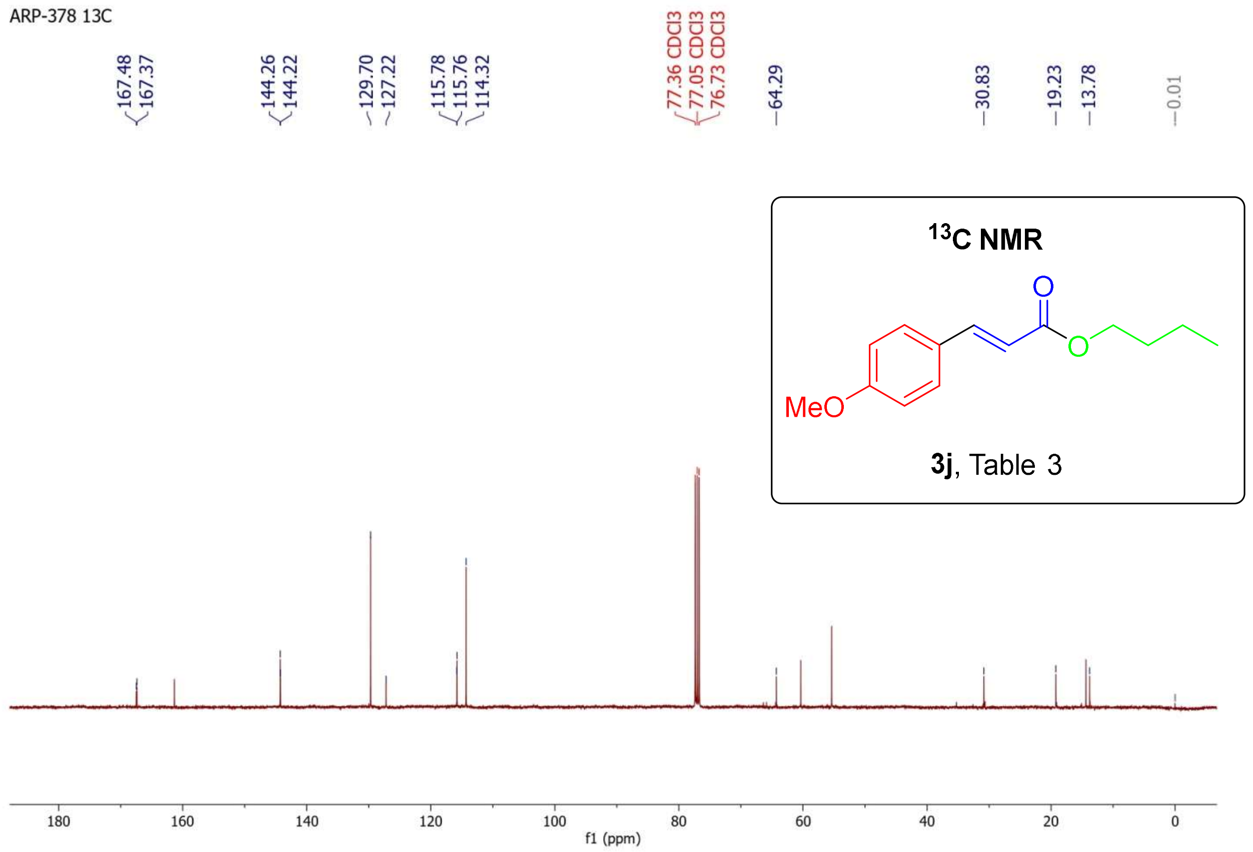

ARP-378 13C
167.48
167.37
144.26
144.22
129.70
127.22
115.78
115.76
114.32
77.36 CDCl3
77.05 CDCl3
76.73 CDCl3
64.29
30.83
19.23
13.78
-0.01
13C NMR
O
MeO
O
3j, Table 3
180
160
140
120
100
80
60
40
20
0
f1 (ppm)

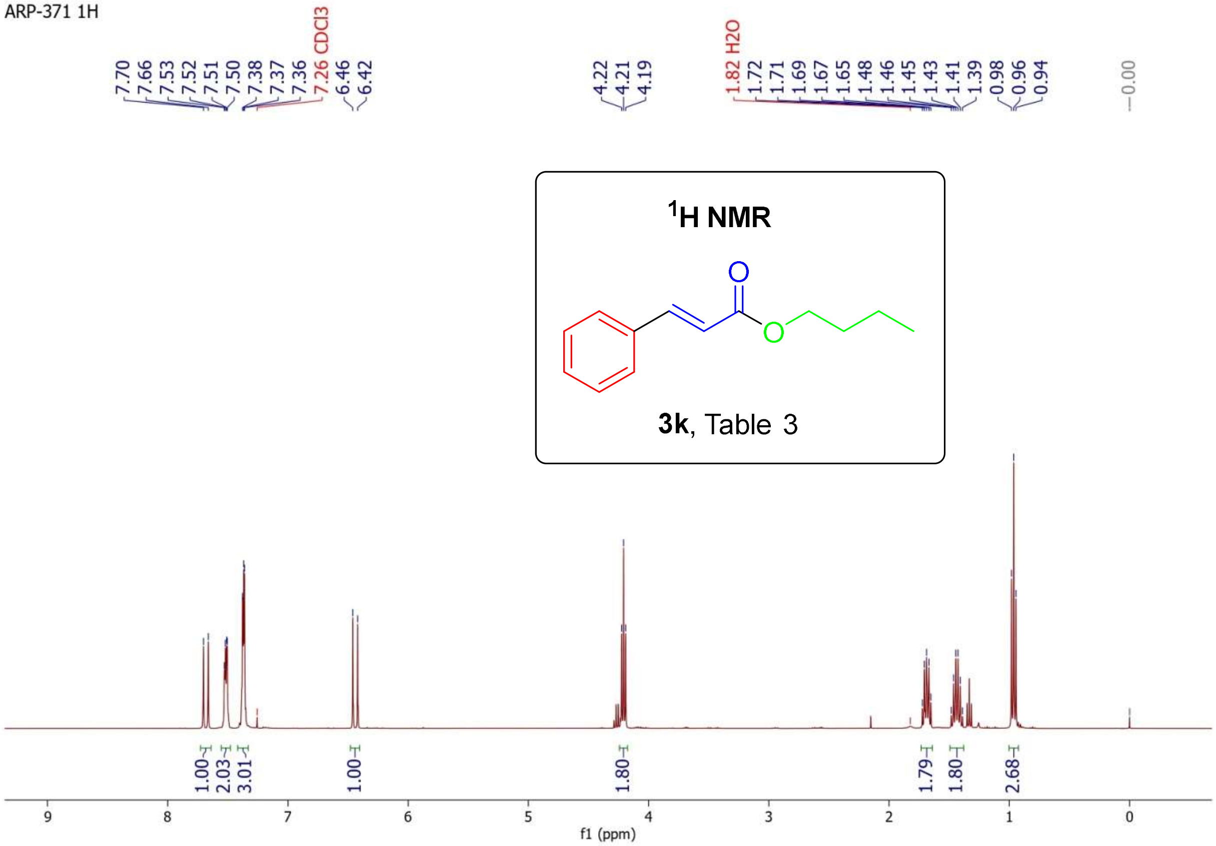
ARP-371 1H
7.70
7.66
7.53
7.52
7.51
7.50
7.38
7.37
7.36
7.26 CDCl3
6.46
6.42
4.22
4.21
4.19
1.82 H2O
1.72
1.71
1.69
1.67
1.65
1.48
1.46
1.45
1.43
1.41
1.39
0.98
0.96
0.94
0.00
1H NMR
O
O
3k, Table 3
1.00
2.03
3.01
1.00
1.80
1.79
1.80
2.68
9
8
7
6
5
4
3
2
1
0
f1 (ppm)

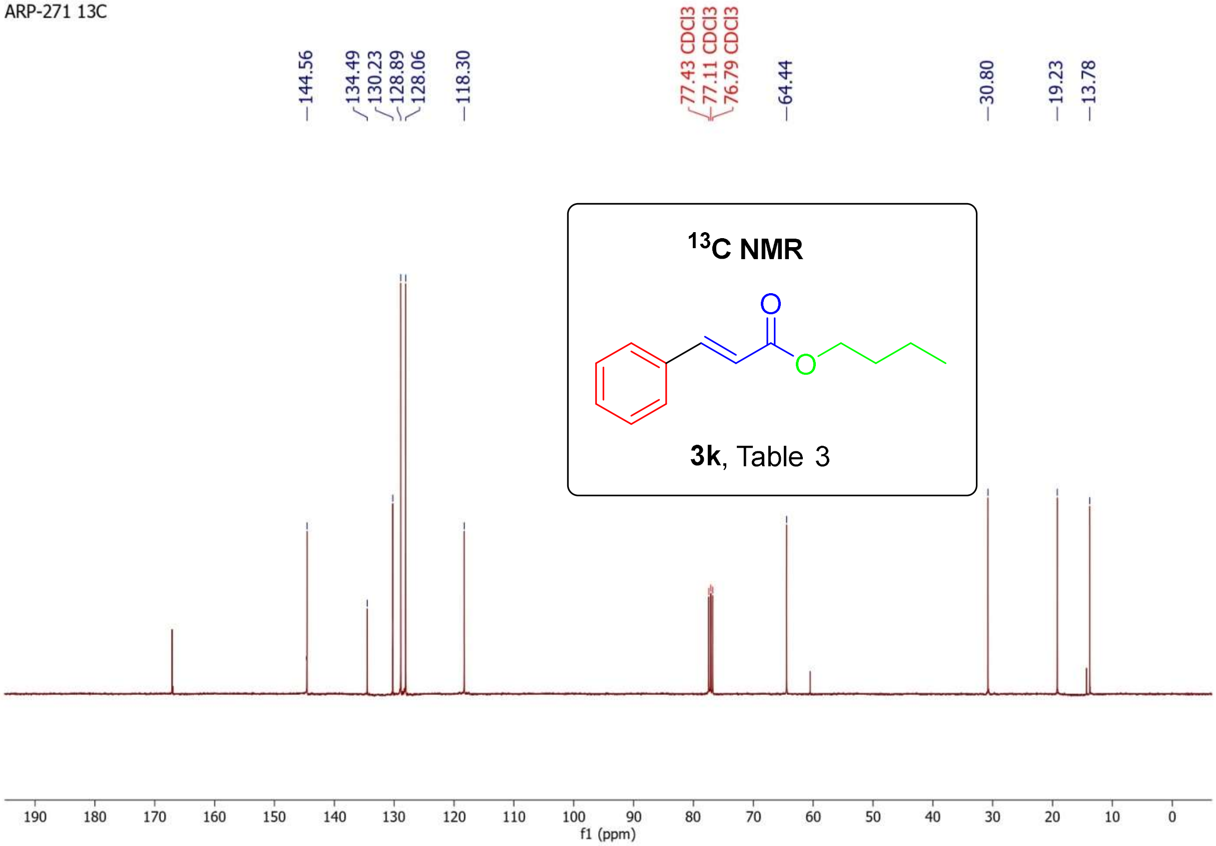
ARP-271 13C
144.56
134.49
130.23
128.89
128.06
118.30
77.43 CDCl3
77.11 CDCl3
76.79 CDCl3
64.44
30.80
19.23
13.78
13C NMR
O
O
3k, Table 3
190 180 170 160 150 140 130 120 110 100 90 80 70 60 50 40 30 20 10 0
f1 (ppm)

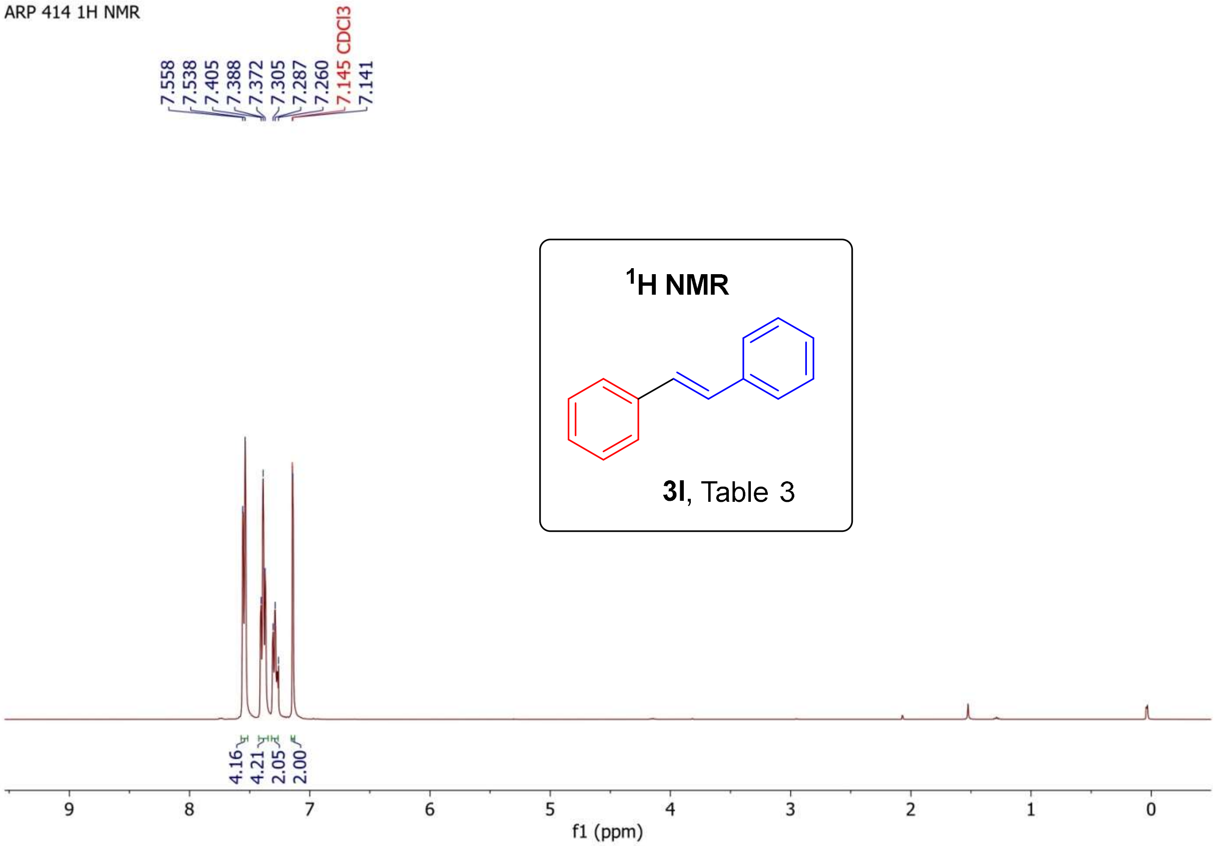
ARP 414 1H NMR
7.558
7.538
7.405
7.388
7.372
7.305
7.287
7.260
7.145 CDCl3
7.141
^{1}H NMR
3l, Table 3
4.16
4.21
2.05
2.00
9
8
7
6
5
4
3
2
1
0
f1 (ppm)

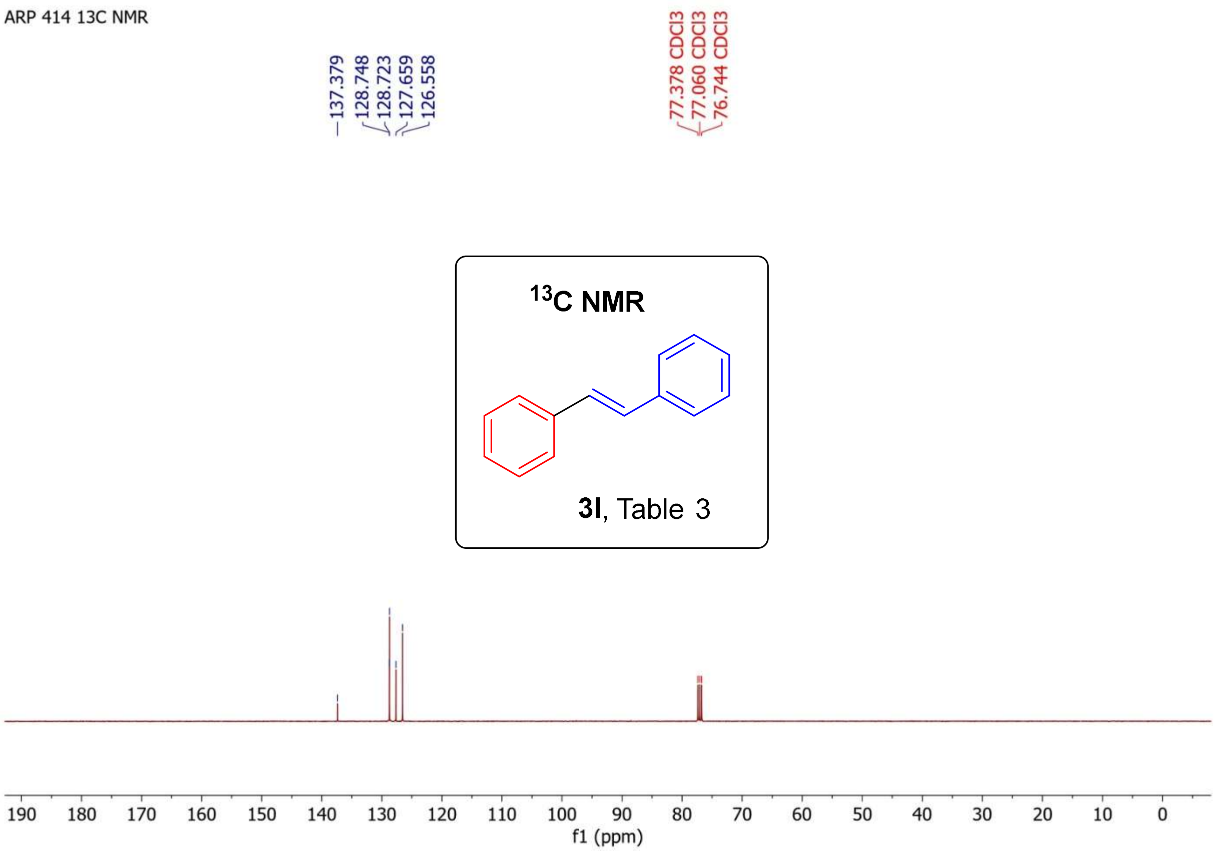
ARP 414 13C NMR
137.379
128.748
128.723
127.659
126.558
77.378 CDCl3
77.060 CDCl3
76.744 CDCl3
13C NMR
3l, Table 3
190
180
170
160
150
140
130
120
110
100
90
80
70
60
50
40
30
20
10
0
f1 (ppm)

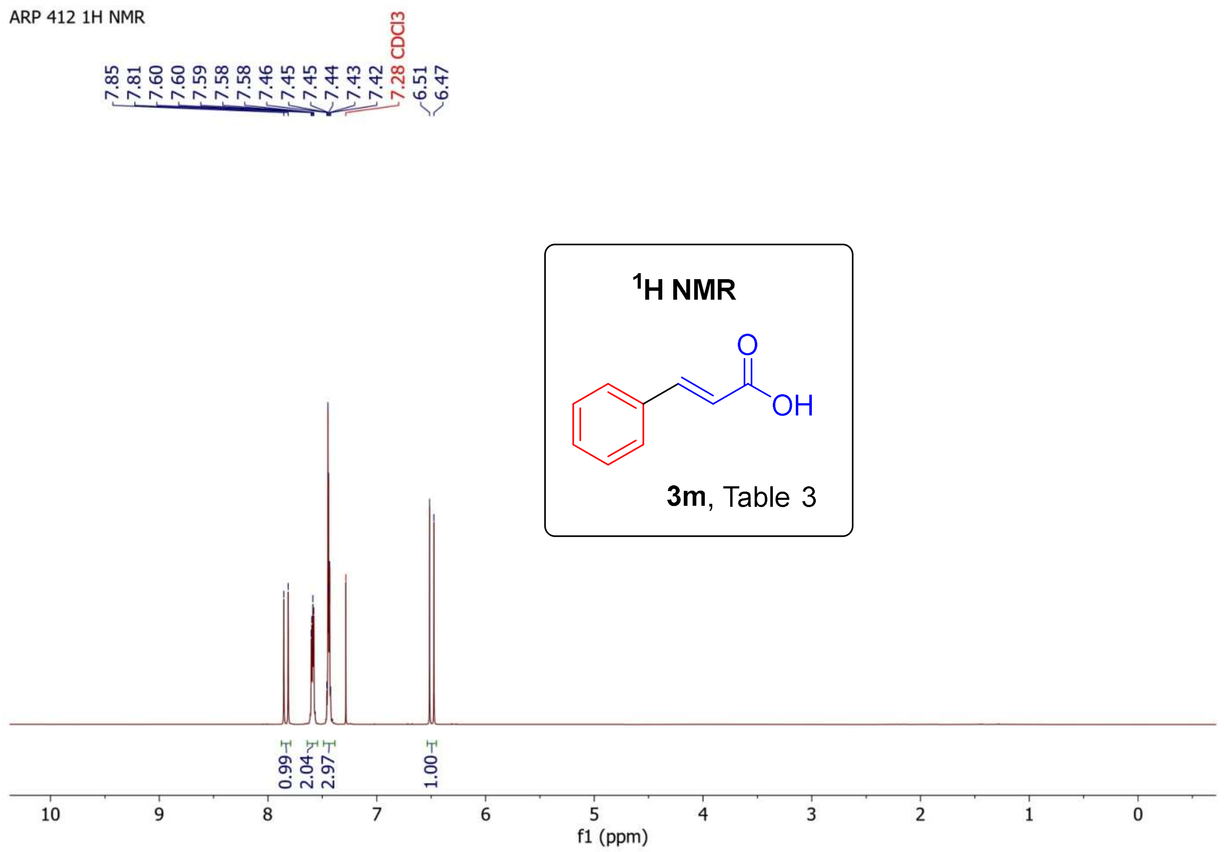
ARP 412 1H NMR
7.85
7.81
7.60
7.60
7.59
7.58
7.58
7.46
7.45
7.45
7.44
7.43
7.42
7.28 CDCl3
6.51
6.47
1H NMR
O
OH
3m, Table 3
0.99
2.04
2.97
1.00
10
9
8
7
6
5
4
3
2
1
0
f1 (ppm)

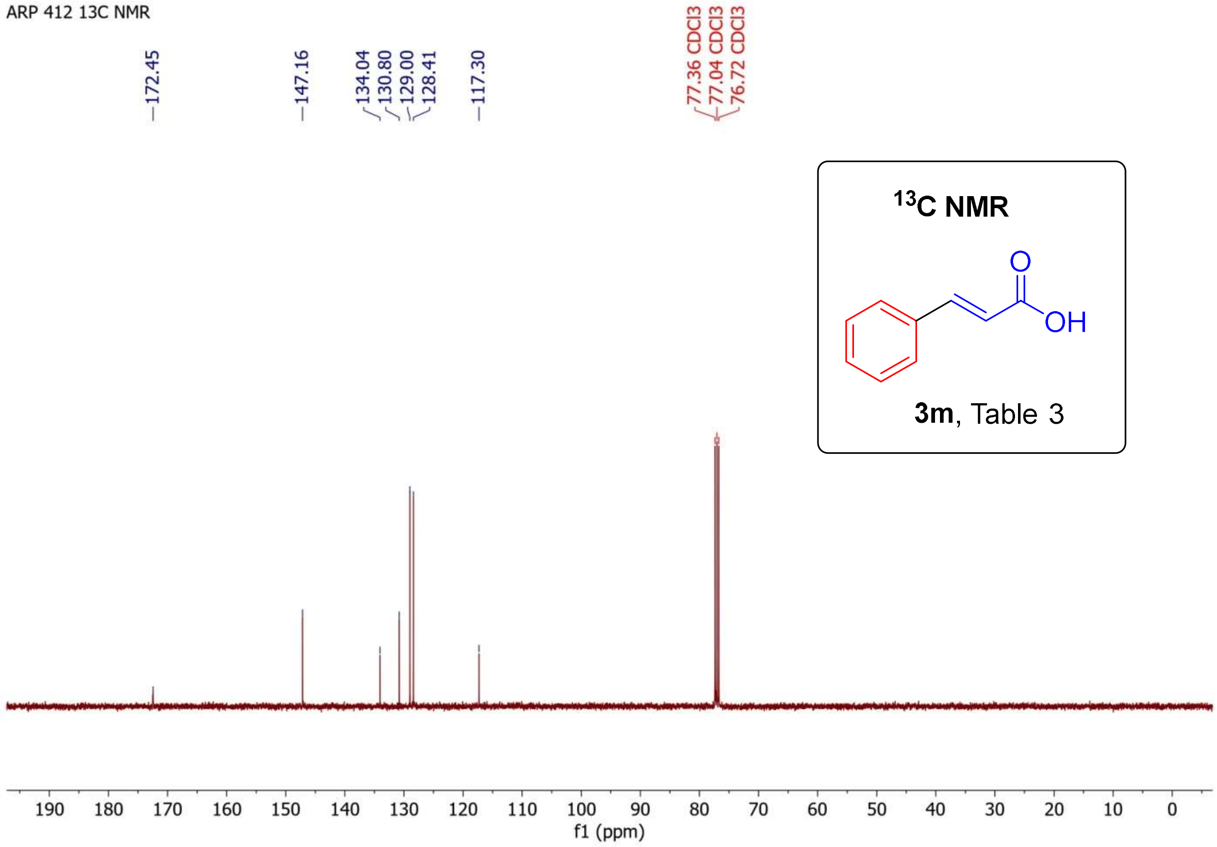

ARP 412 13C NMR
172.45
147.16
134.04
130.80
129.00
128.41
117.30
77.36 CDCl3
77.04 CDCl3
76.72 CDCl3
13C NMR
O
OH
3m, Table 3
190 180 170 160 150 140 130 120 110 100 90 80 70 60 50 40 30 20 10 0
f1 (ppm)

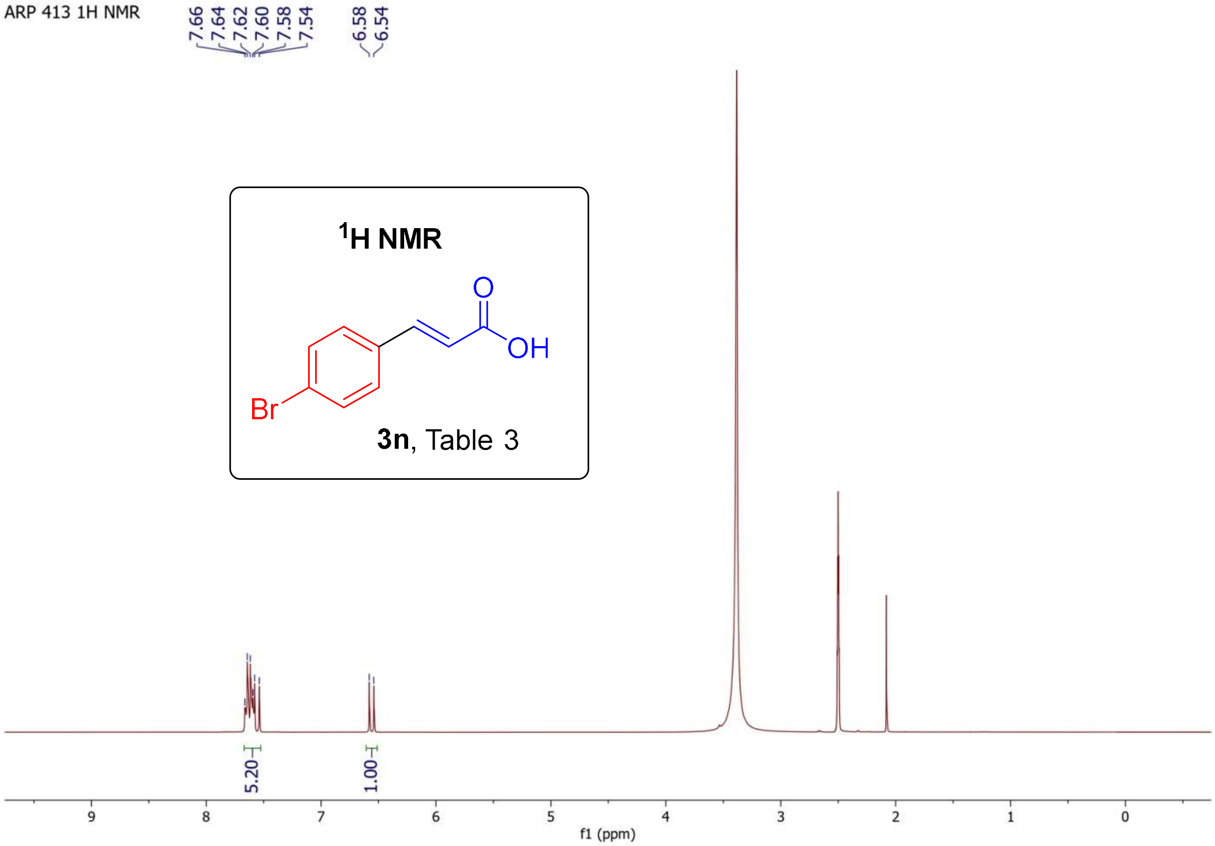
ARP 413 1H NMR
7.66
7.64
7.62
7.60
7.58
7.54
6.58
6.54
1H NMR
O
OH
Br
3n, Table 3
5.20
1.00
9
8
7
6
5
4
3
2
1
0
f1 (ppm)

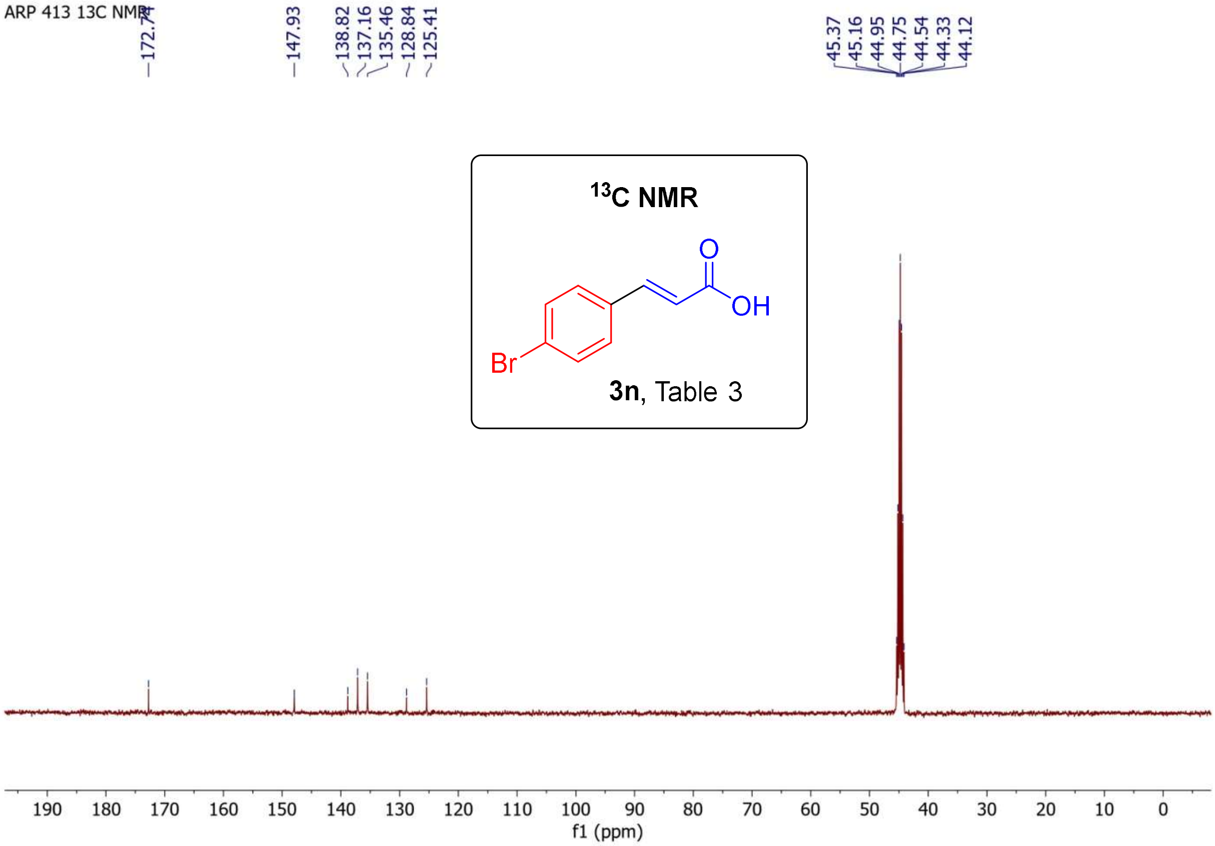
ARP 413 13C NMR
172.74
147.93
138.82
137.16
135.46
128.84
125.41
45.37
45.16
44.95
44.75
44.54
44.33
44.12
13C NMR
O
OH
Br
3n, Table 3
190 180 170 160 150 140 130 120 110 100 90 80 70 60 50 40 30 20 10 0
f1 (ppm)

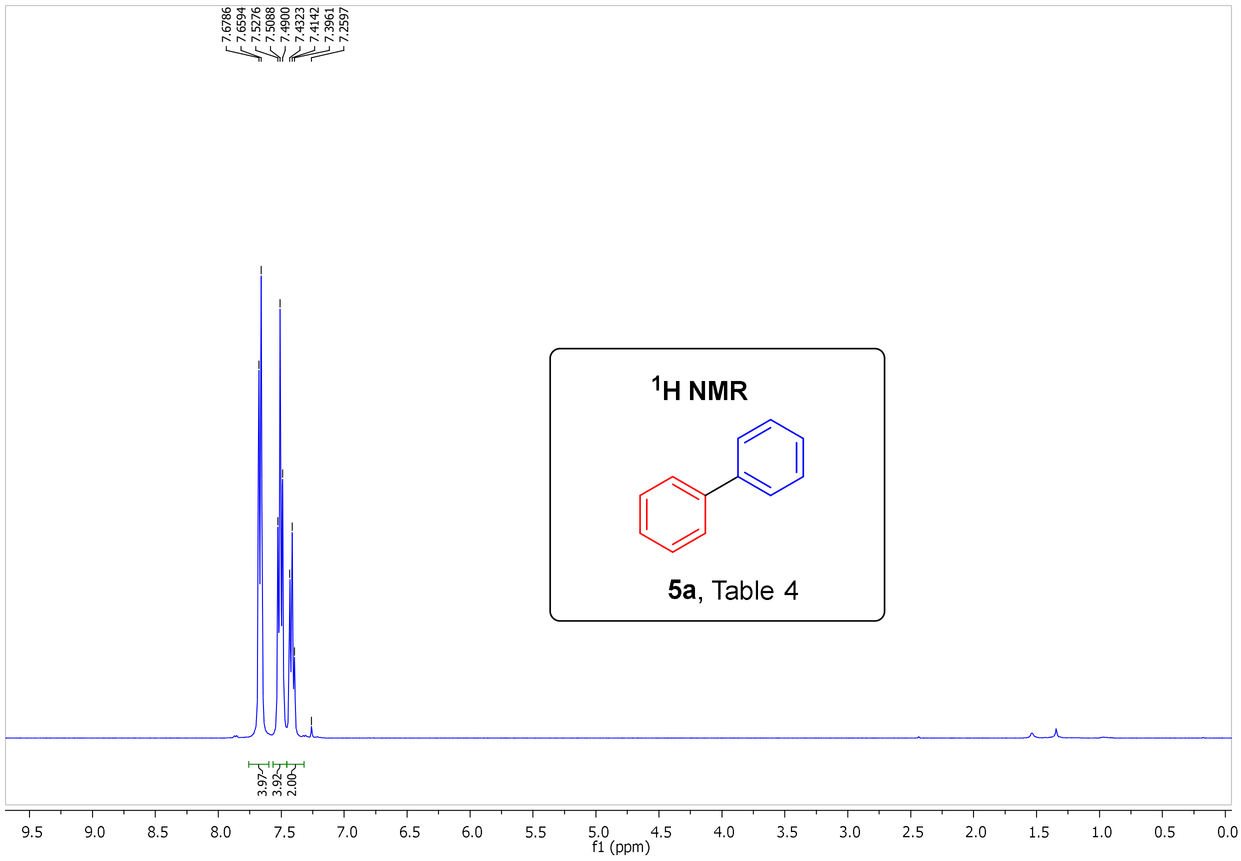

7.6786
7.6594
7.5276
7.5088
7.4900
7.4323
7.4142
7.3961
7.2597
3.97
3.92
2.00
9.5 9.0 8.5 8.0 7.5 7.0 6.5 6.0 5.5 5.0 4.5 4.0 3.5 3.0 2.5 2.0 1.5 1.0 0.5 0.0
f1 (ppm)
1H NMR
5a, Table 4

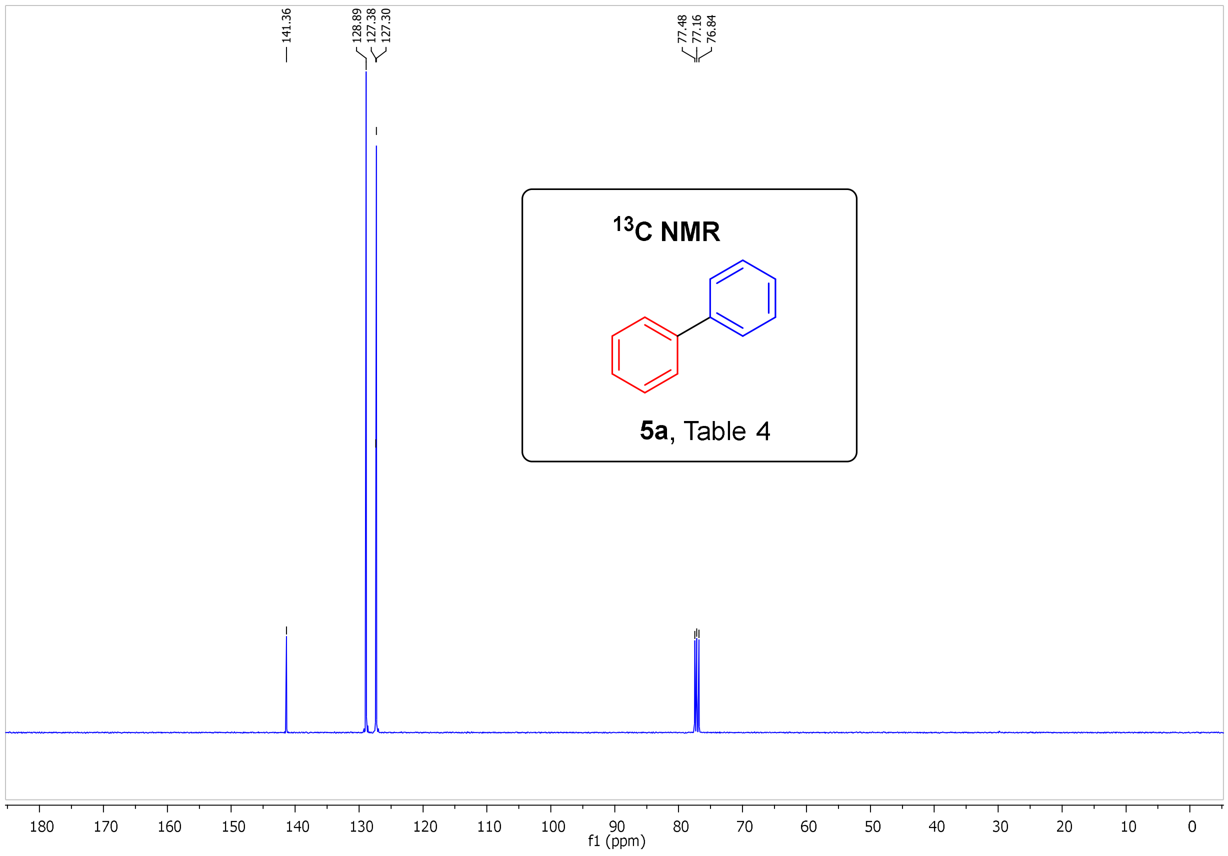
141.36
128.89
127.38
127.30
77.48
77.16
76.84
13C NMR
5a, Table 4
180 170 160 150 140 130 120 110 100 90 80 70 60 50 40 30 20 10 0
f1 (ppm)

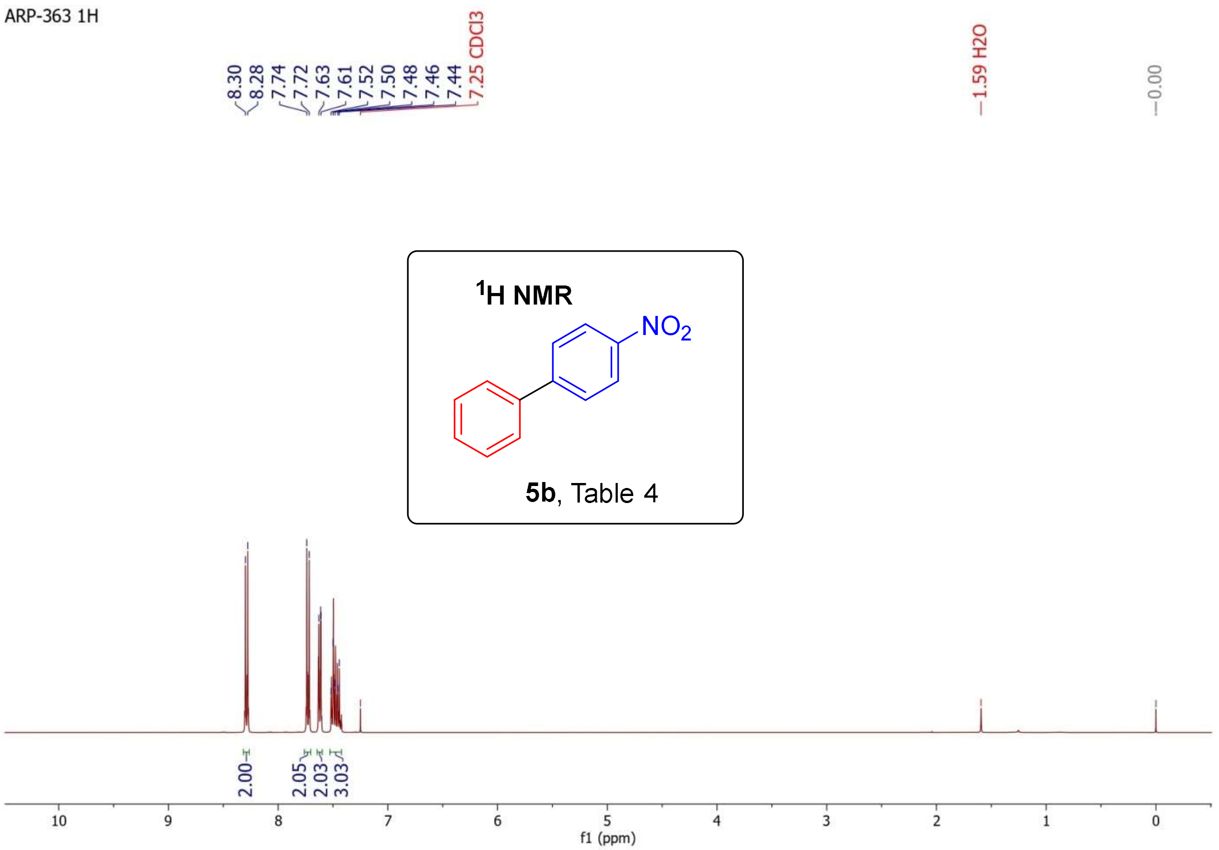
ARP-363 1H
8.30
8.28
7.74
7.72
7.63
7.61
7.52
7.50
7.48
7.46
7.44
7.25 CDCl3
1.59 H2O
0.00
1H NMR
NO2
5b, Table 4
2.00
2.05
2.03
3.03
10
9
8
7
6
5
4
3
2
1
0
f1 (ppm)

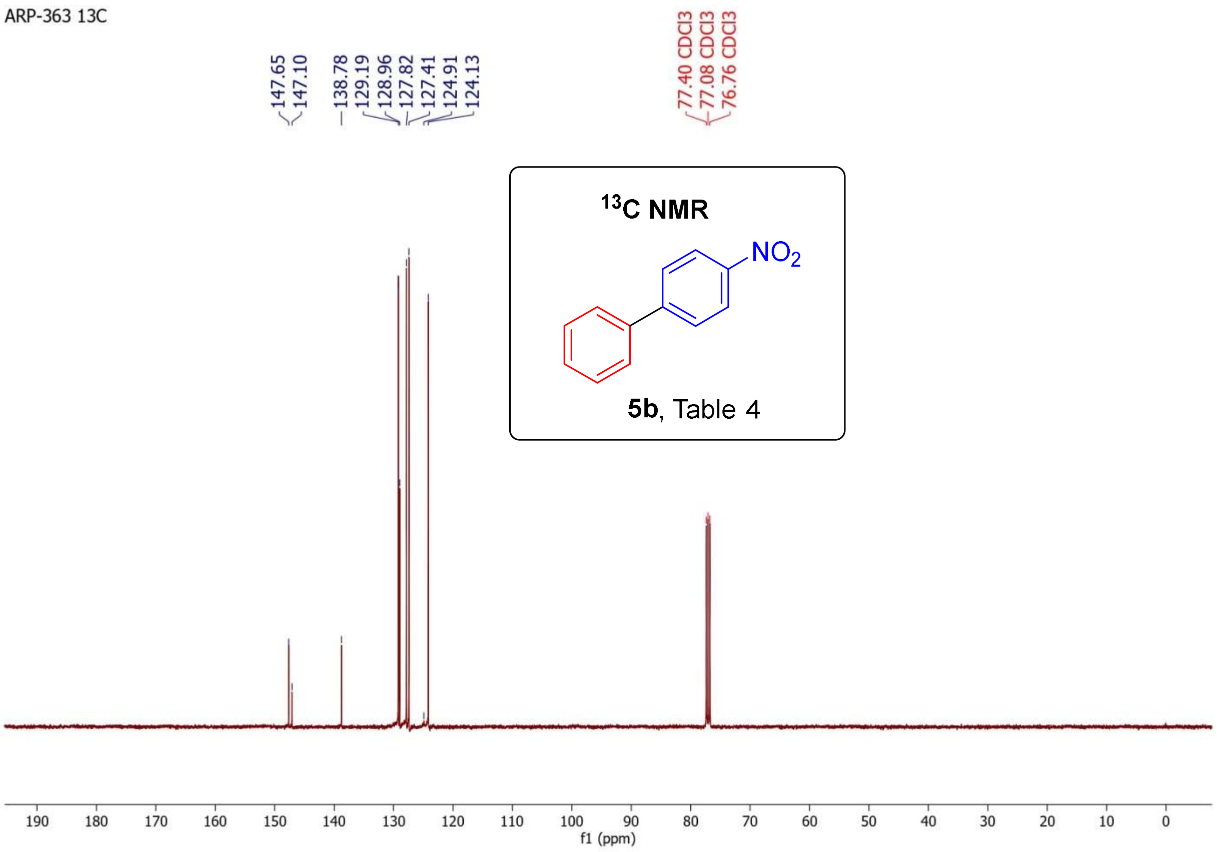
ARP-363 13C
147.65
147.10
138.78
129.19
128.96
127.82
127.41
124.91
124.13
77.40 CDCl3
77.08 CDCl3
76.76 CDCl3
13C NMR
NO2
5b, Table 4
190 180 170 160 150 140 130 120 110 100 90 80 70 60 50 40 30 20 10 0
f1 (ppm)

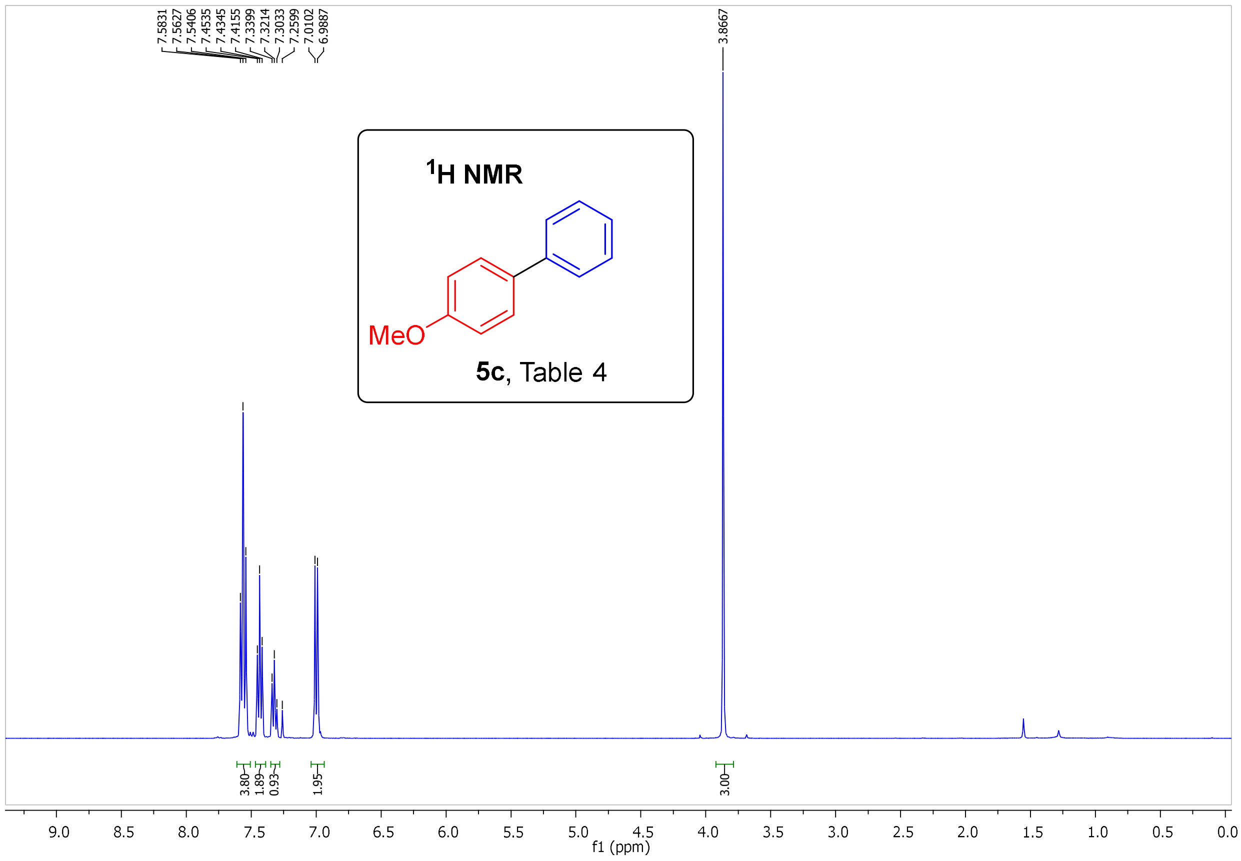
7.5831
7.5627
7.5406
7.4535
7.4345
7.4155
7.3399
7.3214
7.3033
7.2599
7.0102
6.9887
3.8667
1H NMR
MeO
5c, Table 4
3.80
1.89
0.93
1.95
3.00
9.0
8.5
8.0
7.5
7.0
6.5
6.0
5.5
5.0
4.5
4.0
3.5
3.0
2.5
2.0
1.5
1.0
0.5
0.0
f1 (ppm)

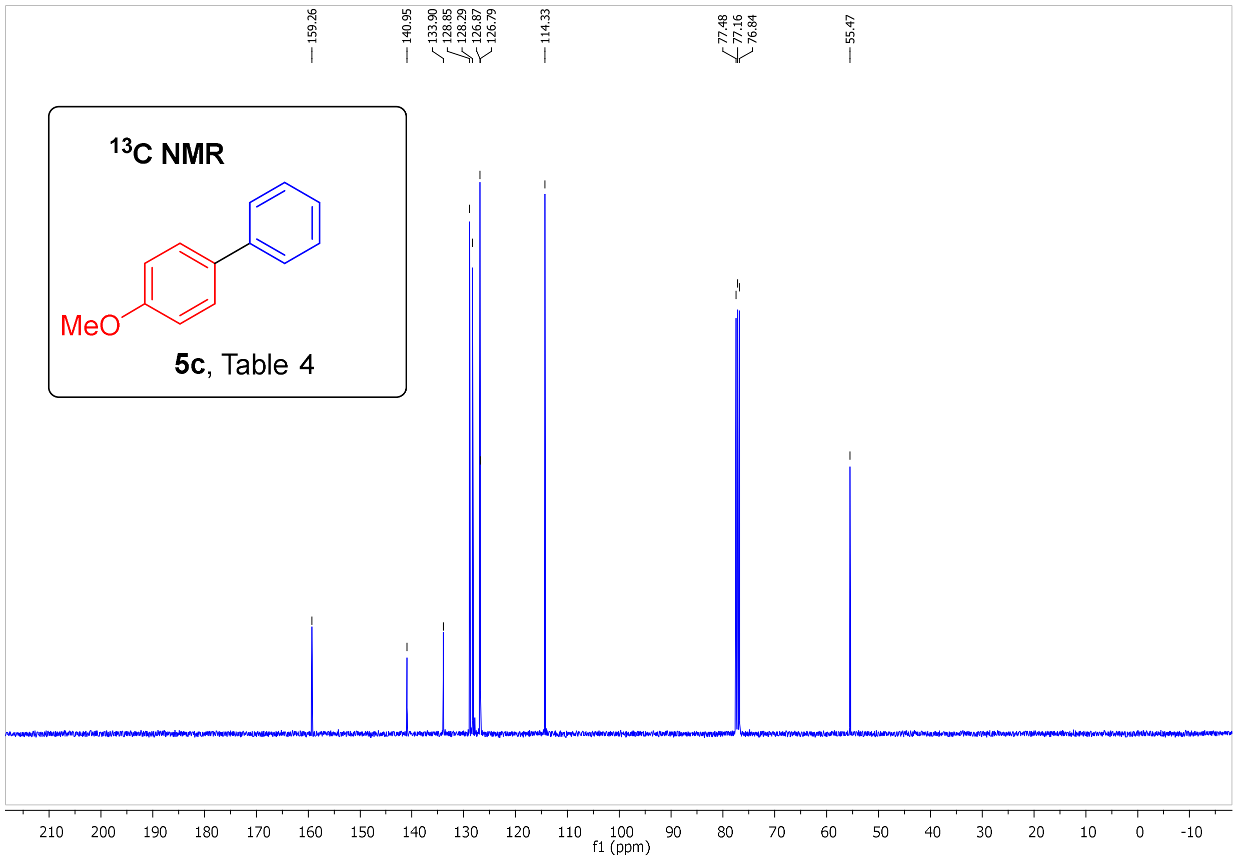

159.26
140.95
133.90
128.85
128.29
126.87
126.79
114.33
77.48
77.16
76.84
55.47
13C NMR
MeO
5c, Table 4
210 200 190 180 170 160 150 140 130 120 110 100 90 80 70 60 50 40 30 20 10 0 -10
f1 (ppm)

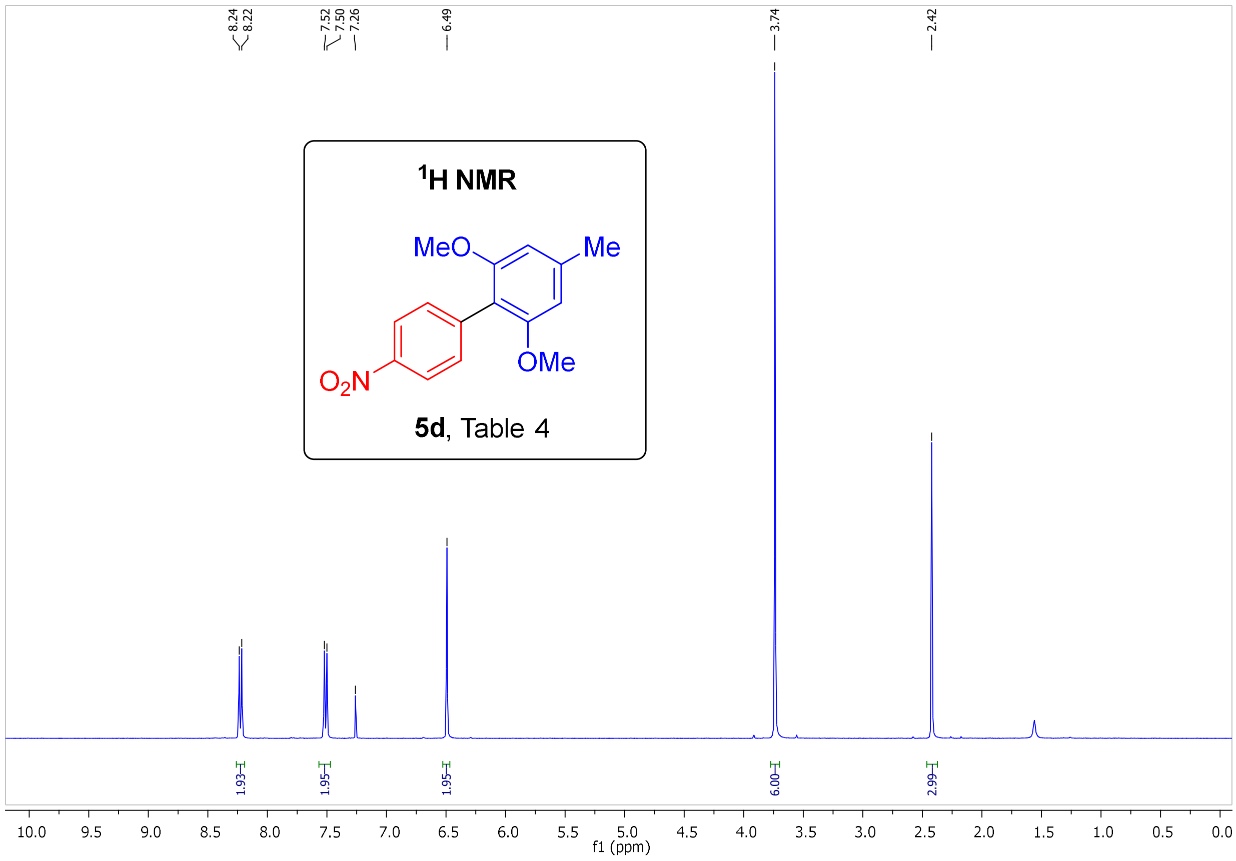
8.24
8.22
7.52
7.50
7.26
6.49
3.74
2.42
1H NMR
MeO
Me
OMe
O2N
5d, Table 4
1.93
1.95
1.95
6.00
2.99
10.0
9.5
9.0
8.5
8.0
7.5
7.0
6.5
6.0
5.5
5.0
4.5
4.0
3.5
3.0
2.5
2.0
1.5
1.0
0.5
0.0
f1 (ppm)

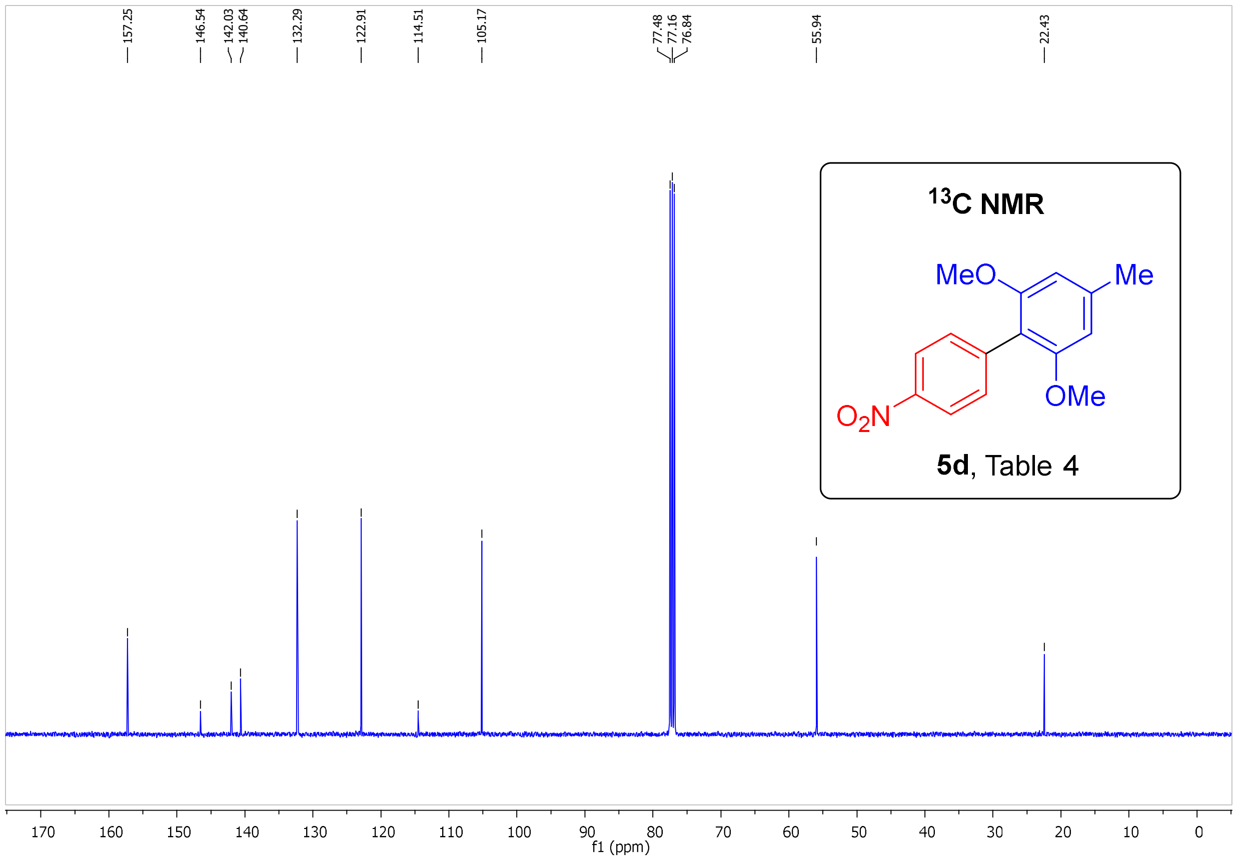
157.25
146.54
142.03
140.64
132.29
122.91
114.51
105.17
77.48
77.16
76.84
55.94
22.43
13C NMR
MeO
Me
OMe
O2N
5d, Table 4
170
160
150
140
130
120
110
100
90
80
70
60
50
40
30
20
10
0
f1 (ppm)

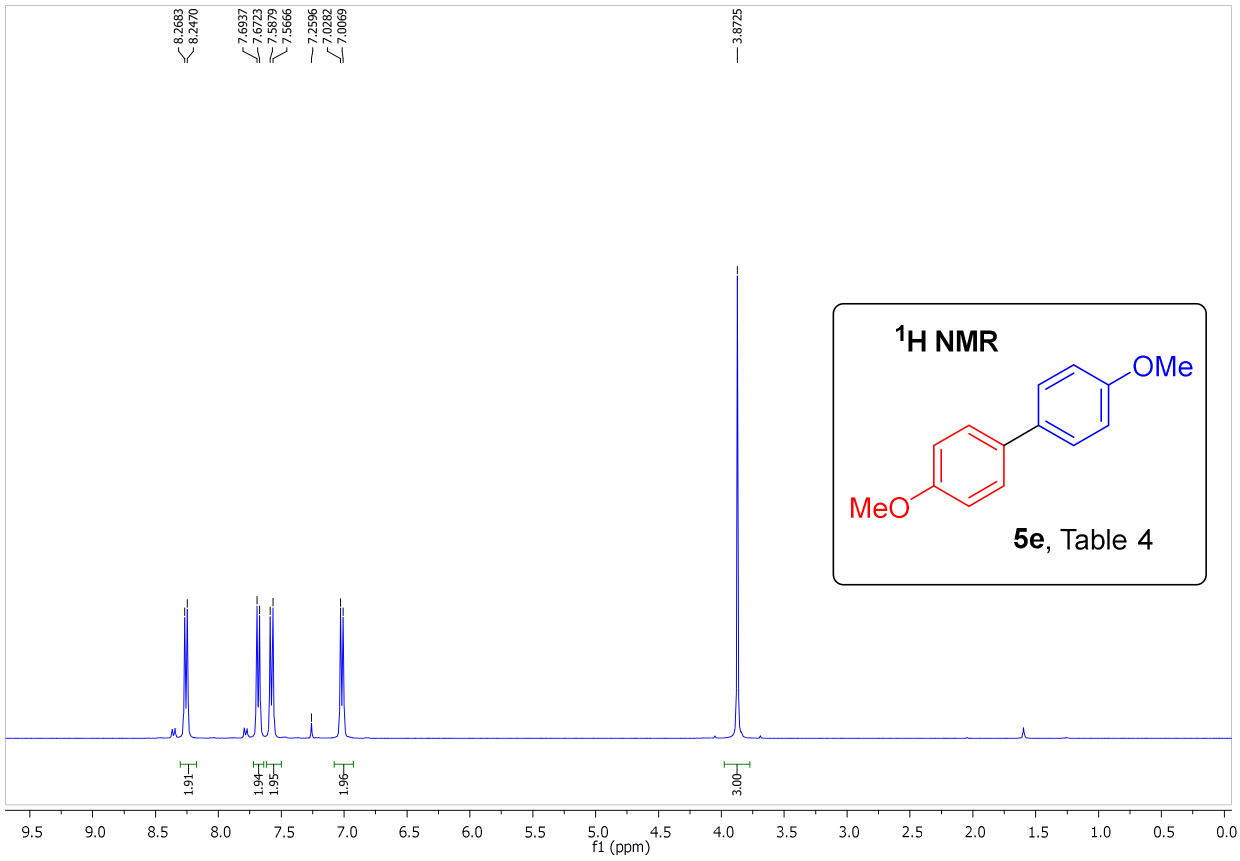
8.2683
8.2470
7.6937
7.6723
7.5879
7.5666
7.2596
7.0282
7.0069
3.8725
1H NMR
OMe
MeO
5e, Table 4
1.91
1.94
1.95
1.96
3.00
9.5
9.0
8.5
8.0
7.5
7.0
6.5
6.0
5.5
5.0
4.5
4.0
3.5
3.0
2.5
2.0
1.5
1.0
0.5
0.0
f1 (ppm)

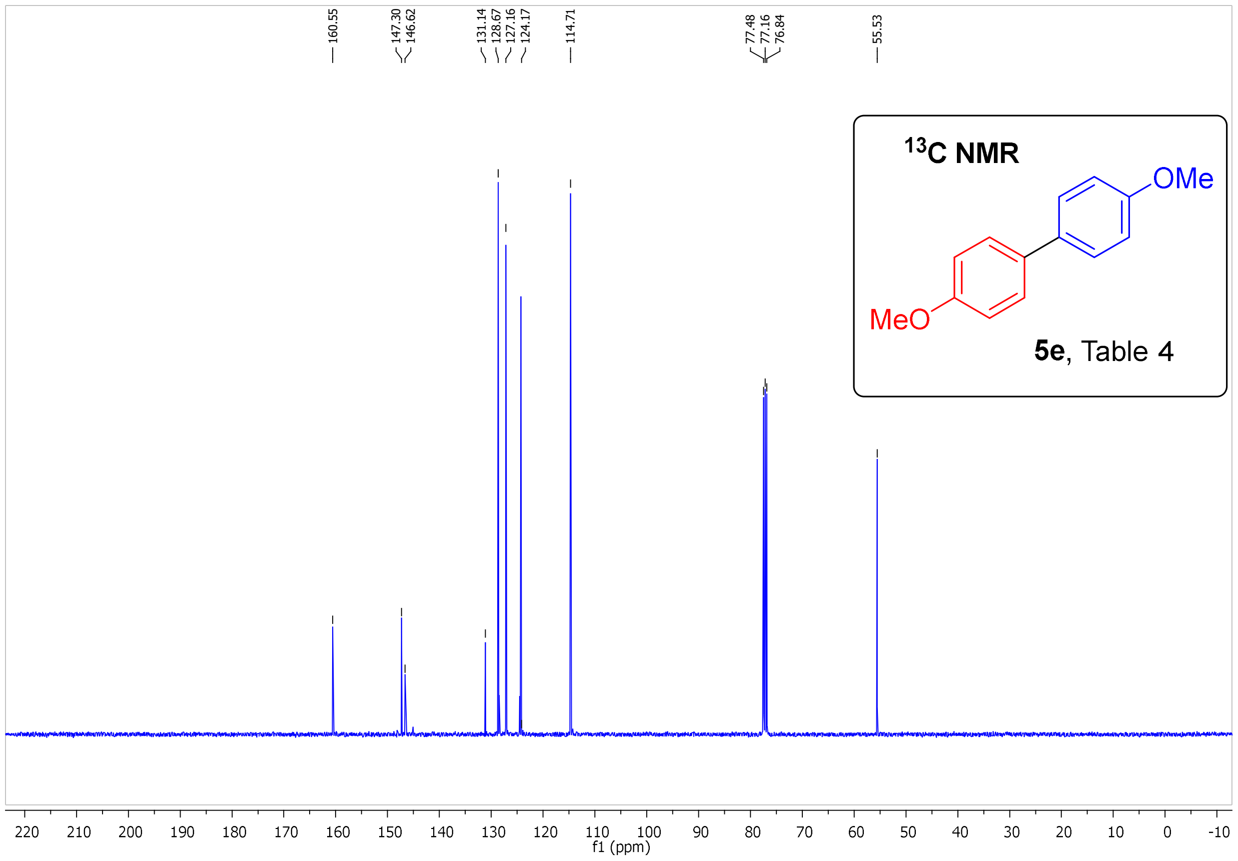
160.55
147.30
146.62
131.14
128.67
127.16
124.17
114.71
77.48
77.16
76.84
55.53
13C NMR
OMe
MeO
5e, Table 4
220
210
200
190
180
170
160
150
140
130
120
110
100
90
80
70
60
50
40
30
20
10
0
-10
f1 (ppm)

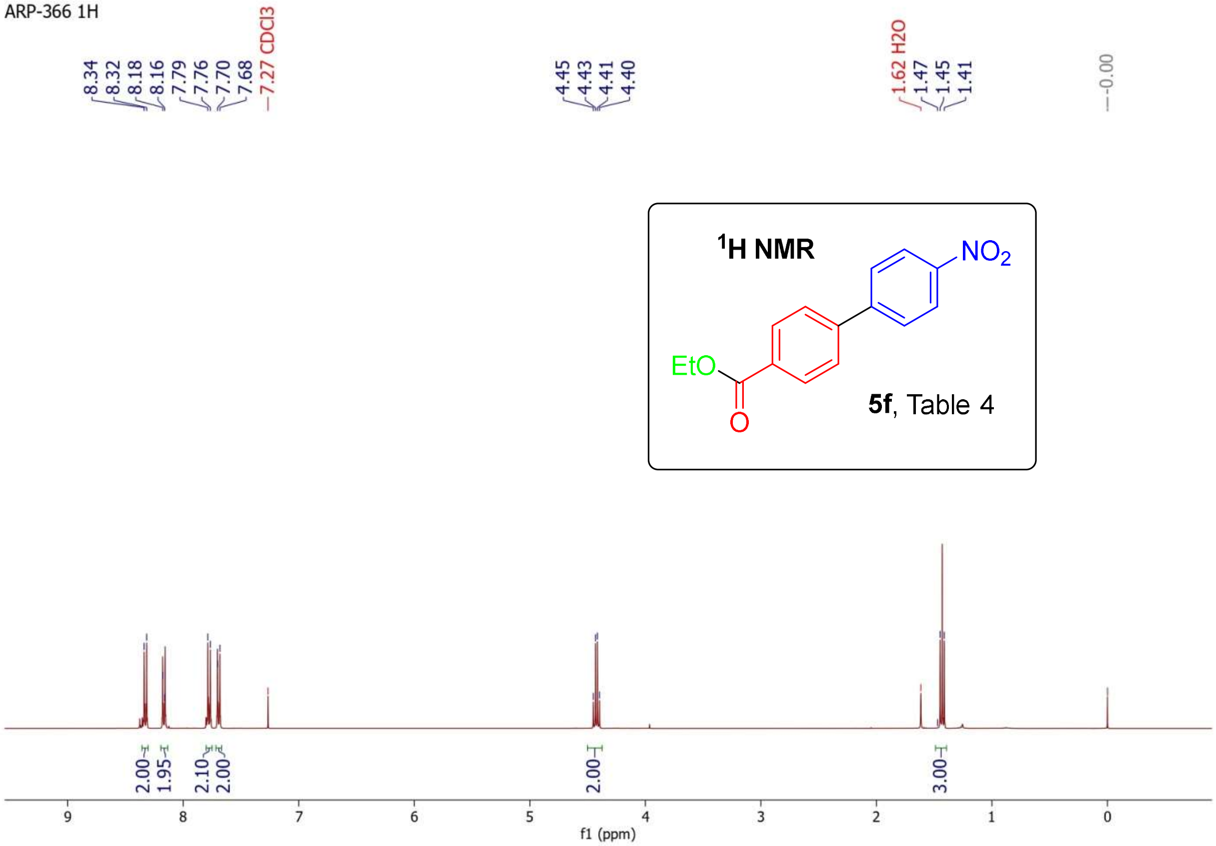

ARP-366 1H
8.34
8.32
8.18
8.16
7.79
7.76
7.70
7.68
7.27 CDCl3
4.45
4.43
4.41
4.40
1.62 H2O
1.47
1.45
1.41
-0.00
1H NMR
NO2
EtO
O
5f, Table 4
2.00
1.95
2.10
2.00
2.00
3.00
9
8
7
6
5
4
3
2
1
0
f1 (ppm)

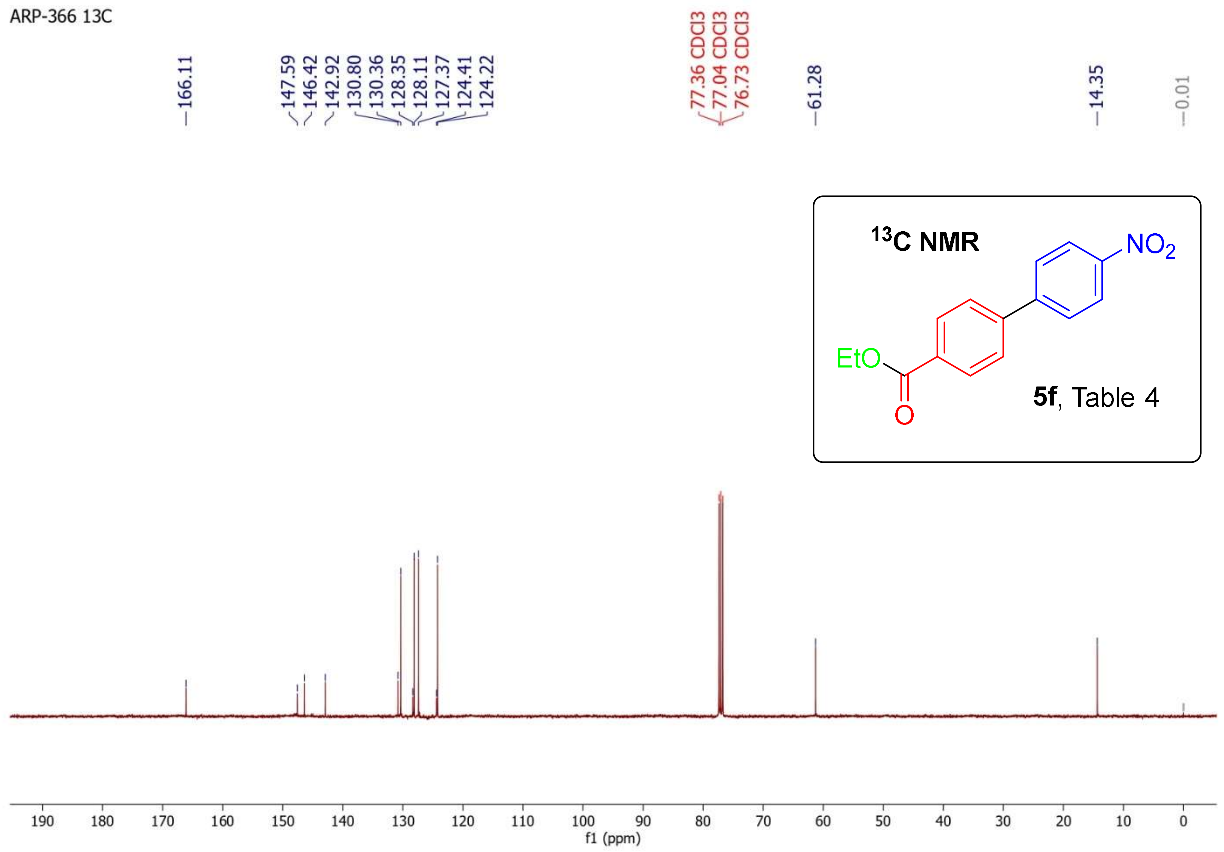

ESI 6. References:

1. Ahuja, B. B.; Sudalai, A., Cu-catalyzed debrominative cyanation of gem-dibromoolefins: a facile access to α, β-unsaturated nitriles. *Organic & Biomolecular Chemistry* **2015,** *13* (21), 5918-5923.

2. Taher, A.; Nandi, D.; Choudhary, M.; Mallick, K., Suzuki coupling reaction in the presence of polymer immobilized palladium nanoparticles: a heterogeneous catalytic pathway. *New Journal of Chemistry* **2015,** *39* (7), 5589-5596.
